# Supplementary material for: Metformin for knee osteoarthritis with obesity: study protocol for a randomised, double-blind, placebo-controlled trial
Source: BMJ Open. 2023 Dec 8;13(12):e079489. doi: 10.1136/bmjopen-2023-079489 (PMC10729261; doi:10.1136/bmjopen-2023-079489)
Supplement: Supplementary data [file bmjopen-2023-079489supp001.pdf]

Participant Information Sheet/Consent Form – Study 1

Monash University

**Title**	Metformin for knee osteoarthritis with obesity – a randomised, double-blind, placebo-controlled trial of a potential disease modifying therapy
**Short Title**	Metformin for Knee Osteoarthritis
**Project Number**	708/20
**Project Sponsor**	Monash University
**Principal Investigators**	Professor Flavia Cicuttini, Dr Yuanyuan Wang, Professor Anita Wluka, Dr Monira Hussain
**Associate Investigators**	Ms Molly Bond, Ms Mahnuma Estee, Dr Yuan Lim, Mr Ashish Dinesh Nair, Dr Benjamin Sutu, Dr Talia Igel, Mr Noor Abid, Dr Rushab Shah, Dr Luigi Zolio, Mr Sehun Daniel Yang
**Location**	School of Public Health and Preventive Medicine, Monash University

Part 1 What does my participation involve?

1 Introduction

You are invited to take part in this research project. This is because you have osteoarthritis in your knee. The research project is testing a new treatment for knee osteoarthritis. The new treatment is metformin, a drug widely used to treat type 2 diabetes that has not been tested in osteoarthritis.

This Participant Information Sheet/Consent Form tells you about the research project. It explains the tests and treatments involved. Knowing what is involved will help you decide if you want to take part in the research.

Please read this information carefully. Ask questions about anything that you don’t understand or want to know more about. Before deciding whether or not to take part, you might want to talk about it with a relative, friend or your local doctor.

Participation in this research is voluntary. If you don’t wish to take part, you don’t have to. You will receive the best possible care whether or not you take part.

If you decide you want to take part in the research project, you will be asked to sign the consent section. By signing it you are telling us that you:

- Understand what you have read
- Consent to take part in the research project
- Consent to have the tests and treatments that are described
- Consent to the use of your personal and health information as described.

You will be given a copy of this Participant Information and Consent Form to keep. The Participant Information and Consent Form is 13 pages long in total. Please make sure that you have all the pages.

Lim YZ, *et al. BMJ Open* 2023; 13:e079489. doi: 10.1136/bmjopen-2023-079489

2 What is the purpose of this research?

Knee osteoarthritis is a common disabling condition causing significant pain and impaired physical function. It is characterised by the loss of cartilage (the lining over the bones that gives a cushioning effect). Currently, there are no treatments that slow knee osteoarthritis progression.

Previous studies have shown that obesity is a significant risk factor for knee osteoarthritis. More than 50% of people with knee osteoarthritis are obese. Evidence suggests that obesity and obesity-associated inflammation and metabolic abnormalities such as diabetes, dyslipidaemia (abnormally elevated lipids or fats in the blood) and hypertension (high blood pressure), are potential treatment targets for those with knee osteoarthritis and obesity.

Metformin is an oral biguanide (a group of oral type 2 diabetes medications that work by preventing the production of sugar in the liver) that is a widely used and effective treatment for patients, including children, with type 2 diabetes mellitus (a condition with abnormally high levels of glucose in the blood). The main effect of metformin is to reduce blood glucose levels, resulting in sustained modest weight loss, and lowering of lipids (or fats) and inflammation. There is emerging evidence suggesting metformin may have the potential to slow the rate of knee cartilage loss and reduce pain in people with knee osteoarthritis and obesity. Metformin is well-tolerated, inexpensive and has a long history of safe clinical use.

The Metformin for Knee Osteoarthritis Study 1 is a randomised clinical trial with the aim to compare the effect of metformin with an inactive 'dummy' tablet (what is called a placebo) on knee pain and function in people with symptomatic knee osteoarthritis and overweight or obesity. If metformin is effective, it will offer a new way to improve pain and function in those with knee osteoarthritis and overweight or obesity.

Metformin has been approved in Australia (by the Australian Government's Therapeutic Goods Administration) to treat type 2 diabetes mellitus for more than 20 years. However, it is not approved to treat knee osteoarthritis. Therefore, it is an experimental treatment for knee osteoarthritis. This means that it must be tested to see if it is an effective treatment for knee osteoarthritis.

The research team conducting this study includes rheumatologists extensively experienced in the treatment of knee osteoarthritis and an endocrinologist extensively experienced with the clinical use of metformin.

A total of 102 participants will participate in this project. They will be randomly allocated into two groups, with one group receiving metformin and the other group receiving a placebo.

This research is a clinical trial being conducted by Monash University. This research has been initiated by the study investigators, Professor Flavia Cicuttini, Dr Yuanyuan Wang, Professor Anita Wluka, and Dr Monira Hussain.

This research has been funded by Monash University.

The results of this research may be used by student investigators to obtain their Honours, Masters or PhD degree.

3 What does participation in this research involve?

If you agree to participate in the study, we will ask you to do the following:

1. Participation in this trial will initially involve a screening process. You will be screened for your suitability for the study over the phone by answering simple questions about your health.

If you meet the inclusion criteria, a Participant Information Sheet will be mailed out to you. You will have an interview with a research assistant and a study doctor through telehealth or onsite study visit at our Clinical Trials Centre (553 St Kilda Road) Monash University, during which any question can be answered. If you agree to participate in the study, you will be asked to provide consent by signing the consent form at the end of this document (either with a pen, if you are at the study site, or electronically, if the visit is conducted by telehealth, via REDCap, a secure web platform).

You will undergo a clinical assessment and complete questionnaires.

In order to further assess your eligibility for the study, you will have a knee X-ray at I-MED Radiology and blood tests at Melbourne Pathology.

During these screening procedures, at any stage, if the researchers identify any reason that would put you at increased risk by being involved in the study, or any reason that would make it inappropriate for you to participate in the study, you will be asked not to continue in the study.

If you use telehealth option for the screening visit, you will be asked to sign the consent form electronically in REDCap (i.e. Research Electronic Data Capture, a secure web platform to capture data for clinical research) which can be accessed on a computer, mobile phone, or tablet. You will open the survey and read through the consent form (which you have already received). When you get to the bottom of the consent form, you will have the opportunity to fill in your information and 'sign' your consent by typing in your name or by utilizing REDCap's 'Signature' field type on the survey. You will select "Next Page" and a read only copy of the consent will be generated that you can review, download, and print. At the bottom of the page you will need to select "I certify that all the information in the document above is correct. I understand that clicking "Submit" will electronically sign the form and that signing this form electronically is the equivalent of signing a physical document." and "Submit". Following the telehealth screening/baseline appointment, the research assistant will post a hard copy of the signed eConsent pdf form to you to ensure you have a copy.

2. If you are eligible to participate, you will be randomly assigned to either the treatment or the control group. The term "randomised" means you will not have a choice regarding which group you are in, and you will have equal chance of receiving the active study medication or placebo.

The treatment group will receive metformin tablets (up to a dose of 2000 milligrams, 2 tablets, once daily) and the control group will receive a placebo (up to 2 tablets, once daily). A placebo is a medication with no active ingredients. It looks like the real thing but is not.

You will initially take 500 milligrams (1 tablet) per day for 2 weeks. Providing you do not experience any adverse effects, the dose will be increased by 500 milligrams (1 tablet) per day every 2 weeks until you are taking 2000 milligrams per day. You will take this dose for the rest of the study. So you will take study medication (metformin or placebo) for 6 months.

Week 1-2: 500mg (1 x 500mg tablet) per day
Week 3-4: 1000mg (2 x 500mg tablets) per day
Week 5-6: 1500mg (3 x 500mg tablets) per day
Week 7-8 and onwards: 2000mg (2 x 1000mg tablets) per day

As this is a "double-blind" trial, neither you nor the study investigator will know which treatment you are receiving until the study is completed. However, in certain circumstances the study investigator may find out which treatment you are receiving.

3. The trial will go for 6 months to examine the effect of metformin on reducing knee pain.

During the trial, you will have 2 study visits [through telehealth or attending our Clinical Trials Centre (553 St Kilda Road)]: at baseline and 6 months. Each study visit will take about 60

Lim YZ, et al. BMJ Open 2023; 13:e079489. doi: 10.1136/bmjopen-2023-079489

minutes. We can provide an attendance certificate signed by the research investigator for each in-person onsite study visit. We will post the study medication to your address after your baseline visit (either onsite or via telehealth).

You will also have monthly telephone interview.

4. The following measurements will occur during the study period.
   - At screening: (1) measurement or self-report of height and weight; (2) knee X-ray; and (3) blood test for kidney and liver function, fasting glucose and lipids, vitamin B12, and inflammatory biomarkers.
   - At baseline: (1) questionnaires (electronic or paper-based surveys) - information about your medical history/conditions, medication, employment, education, smoking, alcohol intake, knee pain and function, hand pain, multi-site pain, absence from paid/unpaid work, physical activity, and quality of life; (2) physical examination - height, weight, and waist circumference (if in-person visit is conducted).
   - Monthly telephone interview from month 1 to 5: knee pain and hand pain.
   - At final (6 month) follow-up: (1) questionnaires (electronic or paper-based surveys) - information about your medical history/conditions, medication, knee pain and function, hand pain, multi-site pain, absence from paid/unpaid work, physical activity, and quality of life; (2) physical examination - height, weight, and waist circumference (if in-person visit is conducted); (3) blood test for kidney and liver function, fasting glucose and lipids, vitamin B12, and inflammatory biomarkers.
   - Safety and adverse events will be recorded over the 6 months, over the phone or via face-to-face interview.
   - Compliance will be assessed by pill count at 6 months. Medication adherence will be monitored over the phone by study staff between study visits.

At baseline and final (6 months) study visits, you will be asked to complete questionnaires online in REDCap (i.e. Research Electronic Data Capture, a secure web platform to capture data for clinical research and create online databases and surveys), facilitated by the research assistant. If you prefer a hard copy, you will be provided with printed questionnaires to complete manually; the research assistant will add the data online.

The following table shows you the procedures that are done at each study visit and telephone interview.

	**Screening**	**Double-blind period**		
	**Screening/ baseline assessment**	**Randomisation**	**1-5 months**	**6 months**
**Informed consent**	X			
**Study visit (telehealth or onsite)**	X			X
**Telephone interview (monthly)**			X	
**Knee X-ray**	X			
**Blood test**	X			X
**Medical history/conditions**	X			X
**Medication**	X			X
**Employment and education**	X			
**Smoking and alcohol**	X			
**Questionnaires**				
Knee VAS	X		X	X
WOMAC	X			X

PainDETECT	X			X
Hand VAS	X		X	X
Multi-site pain	X			X
Absence from paid/unpaid work	X			X
AQoL	X			X
IPAQ	X			X
**Physical examination**				
Height, weight, waist circumference	X			X
**Compliance and safety (adverse events)**			X	X
**Dispense medication**		X		
VAS: visual analogue scale; WOMAC: Western Ontario and McMaster Universities Osteoarthritis Index; AQoL: Assessment of Quality of Life; AQoL: Assessment of Quality of Life; IPAQ: International Physical Activity Questionnaire				

This research project has been designed to make sure the researchers interpret the results in a fair and appropriate way.

There are no additional costs associated with participating in this research project, nor will you be paid. All medication, tests and medical care required as part of the research project will be provided to you free of charge.

You will be reimbursed for any reasonable travel and parking associated with the research project visit (maximum $10 per study visit). If study visits are via telehealth, you will receive a gift voucher ($10) after your baseline and final visits.

It is desirable that your local doctor be advised of your decision to participate in this research project. If you have a local doctor, we will provide a letter to your local doctor informing them of your participation in this research project.

If you choose to withdraw from this study before six months, you will be asked to attend a follow-up visit to allow collection of information regarding your health status (questionnaires and physical examination).

4 What do I have to do?

You will be required to initially take 500 milligrams (1 tablet) per day with your evening meal for 2 weeks. Providing you do not experience any adverse effects, the dose will be increased by 500 milligrams (1 tablet) per day every 2 weeks until you are taking 2000 milligrams per day. You will take this for the rest of the study (2 tablets, 1000 milligrams/tablet). So you will take study medication for a total of 6 months.

You will need to commit to taking the investigational medication regularly. You will need to attend all the study visits.

Whilst involved in this study, you can take your regular medications. We will screen for medications unsuitable for this study. You will need to record all medications and complete a questionnaire at study visits. You may not be able to donate blood while you are in the study.

You should tell your doctor and study personnel if you are taking any other medications, including any that you buy without a prescription from a pharmacy, supermarket or health food shop. Some medications may be affected by metformin or may affect how well metformin works. It is also important not to start taking any other medications during the study without talking to your doctor and research staff. If you start any new medications during the study please tell your doctor and the research staff.

5 Other relevant information about the research project

A total of 102 participants with symptomatic knee osteoarthritis and overweight or obesity, aged over 40 years will be recruited in Melbourne. The project will have two groups, with one group receiving metformin and the other group receiving a placebo. All participants will be followed up over 6 months to examine whether metformin has an effect on symptoms.

The project involves researchers from Monash University.

6 Do I have to take part in this research project?

Participation in any research project is voluntary. If you do not wish to take part, you do not have to. If you decide to take part and later change your mind, you are free to withdraw from the project at any stage.

If you do decide to take part, you will be given this Participant Information and Consent Form to sign and you will be given a copy to keep.

Your decision whether to take part or not to take part, or to take part and then withdraw, will not affect your routine treatment, your relationship with those treating you or your relationship with Monash University.

7 What are the alternatives to participation?

You do not have to take part in this research project to receive treatment. You can see your doctor or health care professional to discuss different treatment options for your knee osteoarthritis, such as physiotherapy and medications for pain relief. Your study doctor will discuss these options with you before you decide whether or not to take part in this research project. You can also discuss the options with your local doctor before deciding whether or not to take part in this research project.

8 What are the possible benefits of taking part?

We cannot guarantee or promise that you will receive any benefits from this research. However, some participants may experience possible benefits, such as improvement in their knee symptoms. If this study shows that metformin is effective in reducing knee pain, it may enable this treatment to be available to more people in the future.

We will inform you of any abnormal findings from knee X-rays and other tests, so that you can then consult with your doctor.

9 What are the possible risks and disadvantages of taking part?

Medical treatments often cause side effects. You may have none, some or all of the effects listed below, and they may be mild, moderate or severe. If you have any of these side effects, or are worried about them, talk with your study doctor. Your study doctor will also be looking out for side effects.

<u>Side effects of metformin</u>
Use of metformin is an established safe and well-tolerated treatment to lower blood glucose. The dose of metformin we use in this study are commonly used for type 2 diabetes mellitus. Metformin may have unwanted side effects in some people.

Most common side effects (affects 1 in 10 to 1 in 100 users) include:

- gastrointestinal symptoms: diarrhoea, nausea, vomiting, abdominal pain, indigestion, loss of appetite
- taste disturbance
- headache

Uncommon or rare side effects (affects 1 in 10,000 users) include:

- skin reactions: erythema, pruritus, urticarial
- lactic acidosis
- decrease of vitamin B12 absorption
- liver function test abnormalities or hepatitis resolving upon metformin discontinuation

Pregnancy risks

The effects of metformin on the unborn child and on the newborn baby are not known. Because of this, it is important that research project participants are not pregnant or breast-feeding and do not become pregnant during the course of the research project. You must not participate in the research if you are pregnant or trying to become pregnant, or breast-feeding.

If you are female and child-bearing is a possibility, you will be required to undergo a pregnancy test prior to commencing the research project.

If you are male, you should not father a child or donate sperm for at least three months after the last dose of study medication.

Both male and female participants are strongly advised to use effective contraception during the course of the research and for a period of three months after completion of the research project. You should discuss methods of effective contraception with your study doctor.

If you do become pregnant whilst participating in the research project, you should advise your study doctor immediately. Your study doctor will withdraw you from the research project and advise on further medical attention should this be necessary. You must not continue in the research if you become pregnant.

You should advise your study doctor if you father a child while participating in the research project. Your study doctor will advise on medical attention for your partner should this be necessary.

Unknown side effects

There may be side effects that the researchers do not expect or do not know about and that may be serious. Tell your study doctor immediately about any new or unusual symptoms that you get.

Many side effects go away shortly after treatment ends. However, sometimes side effects can be serious, long lasting or permanent. If a severe side effect or reaction occurs, your study doctor may need to stop your treatment. Tell the study doctor if you have any problems. Your study doctor will monitor for and discuss the best way of managing any side effects with you, should they occur.

Risks of procedures in this study

This research project involves exposure to a very small amount of radiation. As part of everyday living, everyone is exposed to naturally occurring background radiation and receives a dose of about 2 millisieverts (mSv) each year. The effective dose from this research project is less than 0.01 mSv. At this dose level, no harmful effects of radiation have been demonstrated, as any effect is too small to measure. The risk is believed to be minimal.

Have you been involved in any other research studies that involve radiation? If so, please tell us. Please keep information contained within the Patient Information and Consent Form about

your exposure to radiation in this study, including the radiation dose, for at least five years. You will be required to provide this information to researchers of any future research projects involving exposure to radiation.

Having blood taken may cause some discomfort, bruising, minor infection or bleeding. If this happens, it can be easily treated. Blood samples is collected by a qualified venpuncturist. We endeavour to make the collection process as simple and as stress free as possible.

If you become upset or distressed as a result of your participation in the research, the study doctor will be able to arrange for counselling or other appropriate support. Any counselling or support will be provided by qualified staff who are not members of the research project team.

10 What will happen to my test samples?

This research project involves the collection, testing and analysis of your blood samples.

The collection and testing of your blood sample is a mandatory component of the research. This is done to examine your liver function, kidney function, blood lipids, blood glucose, vitamin B12, and inflammatory biomarkers in order to assess your suitability for the study and monitor the safety and efficacy of the treatment.

Your blood samples will be re-identifiable (i.e. coded, without your name on them), and will be tested at Melbourne Pathology for the proposed blood tests. Blood samples will be destroyed after testing.

11 What if new information arises during this research project?

Sometimes during the course of a research project, new information becomes available about the treatment that is being studied. If this happens, your study doctor will tell you about it and discuss with you whether you want to continue in the research project. If you decide to withdraw, your study doctor will make arrangements for your regular health care to continue. If you decide to continue in the research project you will be asked to sign an updated consent form.

Also, on receiving new information, your study doctor might consider it to be in your best interests to withdraw you from the research project. If this happens, he/she will explain the reasons and arrange for your regular health care to continue.

12 Can I have other treatments during this research project?

Whilst you are participating in this research project, you may not be able to take some or all of the medications or treatments you have been taking for your condition or for other reasons. It is important to tell your study doctor and the study staff about any treatments or medications you may be taking, including over-the-counter medications, vitamins or herbal remedies, acupuncture or other alternative treatments. You should also tell your study doctor about any changes to these during your participation in the research project. Your study doctor will also explain to you which treatments or medications need to be stopped for the time you are involved in the research project.

It may also be necessary for you to take medication during or after the research project to address side effects or symptoms caused by participation in the study that you may have. This will be paid for by the sponsor of the study.

13 What if I withdraw from this research project?

If you decide to withdraw from the project, please notify a member of the research team before you withdraw. This notice will allow that person or the research supervisor to discuss any health risks or special requirements linked to withdrawing.

If you do withdraw your consent during the research project, the study investigator and relevant study staff will not collect additional personal information from you, although personal information already collected will be retained to ensure that the results of the research project can be measured properly and to comply with law. You should be aware that data collected by the study investigator up to the time you withdraw will form part of the research project results. If you do not want them to do this, you must tell them before you join the research project.

14 Could this research project be stopped unexpectedly?

This research project may be stopped unexpectedly for a variety of reasons. These may include reasons such as:

- Unacceptable side effects
- The drug/treatment being shown not to be effective
- The drug/treatment being shown to work and not requiring further testing

15 What happens when the research project ends?

At the completion of the trial, if you wish to know whether you received metformin or the placebo, please contact the research personnel for further information.

We will send you a follow-up letter to inform you of the findings of the study.

Metformin or placebo will be provided to you during the trial for a 6 month period at no cost. Once the trial has finished we will not be able to continue to provide this treatment to you. However, if your doctor is in agreement with continuing the treatment after the trial, he/she can provide you with a prescription for metformin. From this point you will need to cover the cost of your medication.

Part 2 How is the research project being conducted?

16 What will happen to information about me?

By signing the consent form you consent to the study investigator and relevant research staff collecting and using personal information about you for the research project. Any information obtained in connection with this research project that can identify you will remain confidential.

Monash REDCap will be used for collection and storage of data. The REDCap is password protected and access restricted to study investigators. The data we collect or use will be individually identifiable or re-identifiable (i.e. coded which means that the data we collect from you will be directly linked to your study ID number. A study ID number will be allocated to each participant so that their name is not on all data.). All electronic data will be kept in password-protected databases, separate from identifying information. The completed eConsent PDFs are stored in REDCap in a File Repository under “PDF Survey Archive”. Where data is collected on paper, this will be kept in locked filing cabinets with restricted key access, at the School of Public Health and Preventive Medicine, Monash University.

Your knee x-ray will be name-identified in accordance with standard clinical practice, and will be stored securely and indefinitely in the I-MED Radiology database and password-protected databases at the School of Public Health and Preventive Medicine, Monash University. Access to data will be limited to the principal and associate investigators and support staff only. Data

Lim YZ, *et al. BMJ Open* 2023; 13:e079489. doi: 10.1136/bmjopen-2023-079489

transfer will occur so that the final dataset with re-identifiable (i.e. coded) data can be accessed by all the chief investigators of the study. Identifiable information will not be released to anyone outside the research team.

Your information will only be used for the purpose of this research project and it will only be disclosed with your permission, except as required by law.

By signing the consent form you consent to the study investigator using your data collected for this project for extended related research. Your data collected for this project may be combined with data collected from other studies for meta-analyses. In this case only re-identifiable data will be used.

Information from questionnaires and physical examinations will be retained for at least 15 years upon completion of the study. This research project does not involve the establishment of a databank.

It is necessary that your local doctor be advised of your decision to participate in this research project. By signing the consent section, you agree to your local doctor being notified of your decision to participate in this research project.

It is anticipated that the results of this research project will be published and presented in a variety of forums. In any publication, report, or presentation, information will be provided in such a way that you cannot be identified, except with your permission. This confidentiality will be maintained by presenting aggregate data.

Should any sharing of data be considered (e.g. for combining data with other studies), then data sharing will only involve re-identified (i.e. coded) data, i.e. data that is shared will not include identifiable information. In the event where personal information (e.g. name, date of birth) may need to be shared (e.g. data linkage), we will contact you or your guardian for consent for data sharing.

In accordance with relevant Australian and Victorian privacy and other relevant laws, you have the right to request access to your information collected and stored by the research team. You also have the right to request that any information with which you disagree be corrected. Please contact the study team member named at the end of this document if you would like to access your information.

If you are not satisfied with how your personal information has been handled (as laid out in the Privacy Act, 1988), then you can make a complaint to the Office of the Australian Information Commissioner (OAIC). Please refer to http://www.oaic.gov.au/privacy/privacy-complaints for more information.

17 Complaints and compensation

If you suffer any injuries or complications as a result of this research project, you should contact the study team as soon as possible and you will be assisted with arranging appropriate medical treatment. If you are eligible for Medicare, you can receive any medical treatment required to treat the injury or complication, free of charge, as a public patient in any Australian public hospital.

18 Who is organising and funding the research?

This research project is being conducted at Melbourne by Professor Flavia Cicuttini, Dr Yuanyuan Wang, Professor Anita Wluka, and Dr Monira Hussain, and is being funded by Monash University.

By taking part in this research project you agree that data generated from this project may be provided to Monash University. Monash University may directly or indirectly benefit financially from knowledge acquired through analysis of your data.

You will not benefit financially from your involvement in this research project even if, for example, knowledge acquired from analysis of your data prove to be of commercial value to Monash University.

In addition, if knowledge acquired through this research leads to discoveries that are of commercial value to Monash University, the study investigators or their institutions, there will be no financial benefit to you or your family from these discoveries.

No member of the research team will receive a personal financial benefit from your involvement in this research project (other than their ordinary wages).

19 Who has reviewed the research project?

All research in Australia involving humans is reviewed by an independent group of people called a Human Research Ethics Committee (HREC). The ethical aspects of this research project have been approved by the Alfred Hospital Ethics Committee.

This project will be carried out according to the *National Statement on Ethical Conduct in Human Research (2007)*. This statement has been developed to protect the interests of people who agree to participate in human research studies.

20 Further information and who to contact

The person you may need to contact will depend on the nature of your query.

If you want any further information concerning this project or if you have any medical problems which may be related to your involvement in the project (for example, any side effects), you can contact the principal investigator:

Professor Flavia Cicuttini (Monash University): 9903 0158, flavia.cicuttini@monash.edu

Clinical contact person

Name	Professor Flavia Cicuttini	
Position	Head Rheumatology, Alfred Hospital<br>Head Musculoskeletal Unit, Monash University	Alfred Hospital Rheumatology Registrar (outside office hours)
Telephone	9903 0158	9076 2000
Email	flavia.cicuttini@monash.edu	

Project team contact information

Name	
Position	Project officer
Telephone	9903 0553
Email	jointstudy@monash.edu

For matters relating to research at the site at which you are participating, the details of the local site complaints person are:

Position	Complaints Officer (Alfred Health)

Telephone	03 9076 3619
Email	research@alfred.org.au

Complaints contact person

Position	Complaints Officer (Monash University)
Telephone	03 9905 2052
Email	muhrec@monash.edu

If you have any complaints about any aspect of the project, the way it is being conducted or any questions about being a research participant in general, then you may contact:

Reviewing HREC approving this research and HREC Executive Officer details

Reviewing HREC name	Alfred Hospital Ethics Committee
Position	HREC Executive Officer
Telephone	03 9076 3619
Email	research@alfred.org.au

Lim YZ, *et al. BMJ Open* 2023; 13:e079489. doi: 10.1136/bmjopen-2023-079489

Consent Form

**Title**	Metformin for knee osteoarthritis with obesity – a randomised, double-blind, placebo-controlled trial of a potential disease modifying therapy
**Short Title**	Metformin for Knee Osteoarthritis
**Project Number**	708/20
**Project Sponsor**	Monash University
**Principal Investigators**	Professor Flavia Cicuttini, Dr Yuanyuan Wang, Professor Anita Wluka, Dr Monira Hussain
**Associate Investigators**	Ms Molly Bond, Ms Mahnuma Estee, Dr Yuan Lim, Mr Ashish Dinesh Nair, Dr Benjamin Sutu, Dr Talia Igel, Mr Noor Abid, Dr Rushab Shah, Dr Luigi Zolio, Mr Sehun Daniel Yang
**Location**	School of Public Health and Preventive Medicine, Monash University

Consent Agreement

I have read the Participant Information Sheet or someone has read it to me in a language that I understand.

I understand the purposes, procedures and risks of the research described in the project.

I give permission for my doctors, other health professionals, hospitals or laboratories outside this hospital to release information to Monash University concerning my disease and treatment for the purposes of this project. I understand that such information will remain confidential.

I have had an opportunity to ask questions and I am satisfied with the answers I have received.

I freely agree to participate in this research project as described and understand that I am free to withdraw at any time during the study without affecting my future health care.

I understand that I will be given a signed copy of this document to keep.

I understand that, if I decide to discontinue the study treatment, I may be asked to attend follow-up visits to allow collection of information regarding my health status.

❒ eConsent was obtained using telehealth via Monash University REDCap.

Declaration by Participant – for participants who have read the information

Name of Participant (please print) ______________________________

Signature ____________________ Date ____________________

Declaration by Study Doctor/Senior Researcher†

I have given a verbal explanation of the research project, its procedures and risks and I believe that the participant has understood that explanation.

Name of Study Doctor/
Senior Researcher† (please print) ______________________________

Signature ____________________ Date ____________________

† A senior member of the research team must provide the explanation of, and information concerning, the research project. Note: All parties signing the consent section must date their own signature.

Lim YZ, *et al. BMJ Open* 2023; 13:e079489. doi: 10.1136/bmjopen-2023-079489
