# Supplementary material for: Metformin for knee osteoarthritis with obesity: study protocol for a randomised, double-blind, placebo-controlled trial
Source: BMJ Open. 2023 Dec 8;13(12):e079489. doi: 10.1136/bmjopen-2023-079489 (PMC10729261; doi:10.1136/bmjopen-2023-079489)
Supplement: Author's manuscript [file bmjopen-2023-079489.draft_revisions.pdf]

BMJ Open

BMJ Open is committed to open peer review. As part of this commitment we make the peer review history of every article we publish publicly available.

When an article is published we post the peer reviewers' comments and the authors' responses online. We also post the versions of the paper that were used during peer review. These are the versions that the peer review comments apply to.

The versions of the paper that follow are the versions that were submitted during the peer review process. They are not the versions of record or the final published versions. They should not be cited or distributed as the published version of this manuscript.

BMJ Open is an open access journal and the full, final, typeset and author-corrected version of record of the manuscript is available on our site with no access controls, subscription charges or pay-per-view fees (http://bmjopen.bmj.com).

If you have any questions on BMJ Open's open peer review process please email info.bmjopen@bmj.com

BMJ Open

Metformin for knee osteoarthritis with obesity – study protocol for a randomised, double-blind, placebo-controlled trial.

Journal:	*BMJ Open*
Manuscript ID	bmjopen-2023-079489
Article Type:	Protocol
Date Submitted by the Author:	02-Sep-2023
Complete List of Authors:	Lim, Yuan ; Monash University School of Public Health and Preventive Medicine<br>Wang, Yuanyuan; Monash University School of Public Health and Preventive Medicine<br>Urquhart, Donna M.; Monash University School of Public Health and Preventive Medicine<br>Estee, Mahnuma; Monash University School of Public Health and Preventive Medicine<br>Wluka, Anita; Monash University School of Public Health and Preventive Medicine<br>Heritier, Stephane; Monash University School of Public Health and Preventive Medicine<br>Cicuttini, Flavia; Monash University School of Public Health and Preventive Medicine
Keywords:	Obesity, Knee < ORTHOPAEDIC & TRAUMA SURGERY, Randomized Controlled Trial

SCHOLARONE™
Manuscripts

Metformin for knee osteoarthritis with obesity – study protocol for a randomised, double-blind, placebo-controlled trial.

Yuan Z Lim[1]*, Yuanyuan Wang[1]*, Donna M Urquhart[1], Mahnuma Mahfuz Estee[1], Anita E. Wluka[1], Stephane Heritier[1], Flavia M. Cicuttini[1]

* YZL and YW contributed equally

[1] School of Public Health and Preventive Medicine, Monash University, Melbourne, VIC 3004, Australia.

Sources of support:

This study is funded by an Investigator Grant from the National Health and Medical Research Council of Australia (NHMRC, APP 1194829). YZL is the recipient of NHMRC Clinical Postgraduate Scholarship (#1133903) and Royal Australasian College of Physicians Woolcock Scholarship. YW is the recipient of NHMRC Translating Research into Practice Fellowship (APP1168185). DMU is a recipient of a NHMRC/Medical Research Future Fund (MRFF) Career Development Fellowship (Clinical Level 2 #1142809). MME is the recipient of Bangabandhu Science and Technology Fellowship from Ministry of Science and Technology, Government of the People's Republic of Bangladesh. FMC is the recipient of NHMRC Investigator Grant (APP1194829). The funders of the study had no role in the study design and conduct of the study; collection, management, analysis and interpretation of the data; preparation, review, or approval of the manuscript; and decision to submit the manuscript for publication.

Corresponding author: Professor Flavia Cicuttini, School of Public Health and Preventive Medicine, Monash University, 553 St Kilda Road, Melbourne, VIC 3004, Australia

Telephone: +61 3 9903 0158; Email: flavia.cicuttini@monash.edu

For peer review only - http://bmjopen.bmj.com/site/about/guidelines.xhtml

Abstract

Introduction

Over half of the populations with knee osteoarthritis (OA) have obesity. These individuals have many other shared metabolic risk factors. Metformin is a safe, inexpensive, well-tolerated drug that has pleotropic effects, including structural protection, anti-inflammatory and analgesic effects in OA, specifically the knee. The aim of this randomised, double-blind, placebo-controlled trial is to determine whether metformin reduces knee pain over 6 months in individuals with symptomatic knee OA who are overweight or obese.

Methods and analysis

One hundred and two participants with symptomatic knee OA and overweight or obesity will be recruited from the community, and randomly allocated in a 1:1 ratio to receive either metformin 2g or identical placebo daily for 6 months. The primary outcome is reduction of knee pain (assessed by 100mm visual analogue scale) at 6 months. The secondary outcomes include change in knee pain, stiffness, function using Western Ontario and McMaster Universities Osteoarthritis Index, quality of life at 6 months, assessed using OMERACT-OARSI responder criteria. Adverse events will be recorded. The primary analysis will be by intention to treat, including all participants in their randomised groups.

Discussion

This study will provide high-quality evidence to address whether metformin has an analgesic effect over 6 months in individuals with symptomatic knee OA who also have overweight or obesity, with a major clinical and public health importance.

Ethics and dissemination

Ethics approval has been obtained from the Alfred Hospital Ethics Committee (708/20) and Monash University Human Research Ethics Committee (28498). The findings will be disseminated through peer-review publications and conference presentations.

Trial registration

Australian New Zealand Clinical Trials Registry, ACTRN12621000710820, registered 8th June 2021.

Strengths and limitations of this study

For peer review only - http://bmjopen.bmj.com/site/about/guidelines.xhtml

- This study is a randomised, double-blind, placebo-controlled trial.
- This study will provide high-quality evidence to address whether metformin has an analgesic effect over 6 months in individuals with symptomatic knee OA with overweight or obesity.
- The generalisability of this study result will be limited to those without diabetes or those not requiring anti-hyperglycaemic therapy.

Keywords

Metformin, knee osteoarthritis, obesity, pain

For peer review only

For peer review only - http://bmjopen.bmj.com/site/about/guidelines.xhtml

Background

Osteoarthritis (OA) is a leading cause of global disability, resulting in 19 million years lived with disability in 2019[1]. There is no approved disease-modifying treatment for OA to date. With limited effective therapies, end-stage OA is treated with total joint replacement, estimated to cost about $10 billion/year in the United States[2] and over $AUD 1 billion/year in Australia[3]. Despite being a multifactorial disease, management of OA has taken a 'one size fits all' approach without considering the different pathological pathways and OA phenotypes, resulting in poor patient outcomes. One distinctive knee OA phenotype is the obese phenotype[4, 5], mediated by inflammatory and metabolic mechanisms[6]. Over 50% of knee OA patients have obesity[7]. Given obesity and obesity-related metabolic factors (hyperglycaemia, dyslipidaemia, hypertension) are all risk factors for knee OA[6, 8], drugs targeting obesity and its associated inflammatory and metabolic abnormalities have the potential to affect the pathogenesis of knee OA.

Metformin is a safe, inexpensive, well-tolerated oral biguanide, which is not only widely used for treatment of type 2 diabetes for over 60 years, but also has a long history of safe use in non-diabetic populations[9, 10]. Additional to its glucose lowering effects, metformin modulates metabolic factors, resulting in at least 2-3kg of weight loss[11, 12] and reduced inflammation and plasma lipids[9, 10]. A recent systematic review of animal and human studies showed metformin has structural protective, anti-inflammatory and analgesic effect for OA, specifically for the knee[13]. These pleotropic effects of metformin are mainly driven by the activation of AMP-activated protein kinase (AMPK) pathway[14-16]. Hence, metformin has the potential to reduce pain in those with knee OA and overweight or obesity.

Study design

This is a randomised, double-blind, placebo-controlled trial in people with symptomatic knee OA and overweight or obesity, to determine the effect of metformin 2g daily versus placebo on reducing knee pain over 6 months.

Hypothesis and objectives

For peer review only - http://bmjopen.bmj.com/site/about/guidelines.xhtml

It is hypothesised that metformin, compared with placebo, will (1) reduce knee pain (primary hypothesis); (2) improve clinical outcomes (stiffness, function, and health-related quality of life); and that (3) the effect of metformin on knee pain and function will be associated with changes in inflammatory and metabolic biomarkers and/or weight loss. If metformin is proven to be effective, it will provide a safe, low-cost treatment to reduce pain and improve function for people with symptomatic knee OA with concurrent overweight or obesity.

Trial registration and reporting

The trial was registered at the Australian New Zealand Clinical Trials Registry prior to commencing recruitment (ACTRN12621000710820, registered 8th June 2021). The trial reporting will be guided by the Consolidated Standards of Reporting Trials (CONSORT) Statement[17].

Ethics approval

Ethics approval has been obtained from the Alfred Hospital Ethics Committee (708/20) and Monash University Human Research Ethics Committee (28498). Written informed consent will be obtained from all the participants.

Methods

Study setting and participants

Participants with symptomatic knee OA and overweight or obesity will be recruited using a combined strategy including collaboration with medical practitioners and advertisements in social and local media.

Inclusion criteria

(1) Men and women aged >40 years, with overweight or obesity (body mass index ≥25 kg/m^2); (2) Knee pain for at least 6 months with a pain score ≥40 mm on a 100 mm visual analogue scale (VAS); (3) Meet the American College of Rheumatology clinical criteria for knee OA[18].

For peer review only - http://bmjopen.bmj.com/site/about/guidelines.xhtml

Exclusion criteria

(1) Severe radiographic knee OA (Kellgren-Lawrence grade 4) or severe knee pain (on standing >80 mm on a 100 mm VAS); (2) Any inflammatory arthritis or significant knee injury; (3) Known or newly diagnosed diabetes requiring anti-hyperglycemic therapy or previous adverse reaction to metformin; (4) Index knee surgery (arthroscopy or open surgery) in the past year; (5) Index knee intra-articular hyaluronic acid injection in the past 6 months or corticosteroid injection in the past 3 months; (6) Use of any investigational drugs or device within 30 days prior to randomisation; (7) Index knee planned joint replacement or arthroscopy in the next 6 months; (8) Other muscular, joint or neurological condition affecting lower limb function; (9) Acute or chronic renal or liver impairment; (10) Other medical condition precluding study participation or relocation; (11) Women who are pregnant, lactating or trying to become pregnant. Use of menopausal hormone therapy or oral contraceptive pill will be permitted so long as the dose has been stable for at least 30 days prior to study entry.

Study timeline

This trial began recruitment on 16th June 2021. It is estimated to finish recruitment on 30th September 2023 and complete the 6-month follow-up and data collection in March 2024. Figure 1 shows trial participation and study procedure.

Randomisation, allocation concealment and blinding

Allocation of participants in a 1:1 ratio to one of the two groups will be by computer generated random numbers prepared by a statistician with no involvement in the trial. Randomisation based on blocks of size 4 and 6 will be performed. The use of a central automated allocation procedure with security in place will ensure the allocation cannot be accessed or influenced by any person. Allocation concealment and double blinding will be ensured by: (1) medications being dispensed by Syntro Pharmacy Pty Ltd; (2) use of an identical placebo tablet; (3) subjective measures being taken by research assistants blinded to group allocation. Participants, assessors and statisticians will be blinded to group allocation.

Intervention and dosing

For peer review only - http://bmjopen.bmj.com/site/about/guidelines.xhtml

All participants will undergo usual care by their treating health practitioners. Eligible participants will be randomly assigned to receive either metformin (up to 2000 mg) once daily or placebo once daily. Treatment with study drug will be initiated at a dose of 500 mg once a day with the evening meal. Over 6 weeks, the dose of study drug will be titrated to 2000 mg once daily (or placebo once daily) to minimise gastrointestinal side effects.

Safety

Any adverse events or serious adverse event will be reported during the study. Blood tests will occur at screening. No data safety monitoring board is required as this agent is approved by Therapeutic Goods Administration with well-known safety profile[19]. Unblinding participants due to side effects of metformin was not an issue in a previous clinical trial[12].

Compliance

Compliance with trial medication will be assessed at 6 months by pill count. Study staff will phone participants in the middle between study visits to monitor medication adherence. Monthly telephone contact for the first 5 months will be conducted to address any concerns, as well as following up knee pain outcome (VAS). This will help to mitigate non-compliance.

Concomitant medication

To maintain the pragmatic nature of the trial, there are no restrictions with regards to concomitant medications, including glucosamine, chondroitin, non-steroidal anti-inflammatory drugs, and opioids analgesics, which will be allowed during the trial and be recorded by questionnaire at all visits. Patients will be asked to keep medications as stable as possible and use paracetamol as rescue medication.

Study procedure

Participants will be screened via phone by questionnaire before attending the screening visit via telehealth. There will be 2 study visits (onsite or telehealth): screening/baseline and month 6, as shown in Table 1. At screening, participants will complete questionnaires, have a knee X-ray and blood tests

For peer review only - http://bmjopen.bmj.com/site/about/guidelines.xhtml

[renal and liver function, plasma glucose and lipids, vitamin B12, and inflammatory biomarkers (C-reactive protein, tumour necrosis factor, interleukin-1 and interleukin 6)] to ensure inclusion criteria are met and exclusion criteria are absent. The index knee will be defined as having symptomatic OA. If both knees are symptomatic and eligible based on VAS pain, the one with higher VAS pain score will be used; if both knees are symptomatic with the same pain level, the one with least severe radiographic OA (joint space narrowing) will be used; if both knees have the same pain level and radiographic severity of OA, the dominant knee will be used. Physical examinations and questionnaires will be performed at months 0 and 6. The same researchers, blinded to treatment allocation, will measure all clinical variables, administer questionnaires, monitor compliance, and record adverse events. Participants are able to withdraw at any time during the trial; the time and reasons will be recorded. If participants withdraw from the study, they will be requested to complete questionnaires (posted to the participants with return envelope).

Primary outcome

The primary outcome is pain reduction at 6 months, measured by change in VAS knee pain from baseline to 6 months (follow-up VAS pain score - baseline score). Knee pain will be measured at baseline and monthly follow-up using a 100 mm VAS by asking “on this line, where would you rate your knee pain over the last 7 days?” with terminal descriptors ‘no pain’ (score 0) and ‘worst imaginable pain’ (score 100).

Secondary outcomes

OMERACT-OARSI responder criteria

This will be used to define a responder based on improvement in WOMAC pain and function and the participant's global assessment[20] at 6 months. Participant's global assessment will be evaluated by 100-mm VAS[21].

Change in knee pain, stiffness and function

Knee pain, stiffness and function will be assessed using the Western Ontario and McMaster Universities Osteoarthritis Index (WOMAC)[22] at baseline and 6 months.

For peer review only - http://bmjopen.bmj.com/site/about/guidelines.xhtml

For peer review only

Health-related quality of life

This will be measured using the Assessment of Quality of Life (AQoL)[23] at baseline and 6 months.

Other measures

Descriptive data: Data regarding age, gender, height, weight, duration of symptoms, employment, medical history, medication use, education level, smoking, alcohol consumption will be collected using a questionnaire at baseline.

PainDETECT: PainDETECT is a validated questionnaire used to assess pain sensitisation in OA[24], and will be assessed at baseline and 6 months.

Physical activity: Physical activity will be measured using the International Physical Activity Questionnaire (IPAQ)[25] short version at baseline and 6 months.

Hand VAS: Pain reduction in hands will be measured at baseline, then monthly for 6 months, according to the OARSI recommendations for the design and conduct of clinical trials for hand OA, which recommend the use of single question pain VAS[26].

Multi-site pain: The presence and levels of multi-site musculoskeletal pain will be assessed at baseline and 6 months using a questionnaire.

Adverse events: These will be measured in a log-book by the blinded assessor at each follow-up.

Biochemical parameters: General (cell counts, liver and renal function), inflammatory biomarkers (C-reactive protein, erythrocyte sedimentation rate, interleukin-6, tumour necrosis factor), plasma glucose and lipids, and vitamin B12 will be measured at baseline and 6 months.

Knee X-ray

X-ray of the study knee (weight-bearing anteroposterior view) will be scored using Kellgren-Lawrence grade. Our intra- and inter-observer reliability is 0.93 and 0.86 for osteophytes, 0.93 and 0.85 for joint space narrowing, respectively[27].

Sample size calculation

The primary outcome is change in VAS knee pain over 6 months. The mean VAS pain was 55 mm (out

For peer review only - http://bmjopen.bmj.com/site/about/guidelines.xhtml

For peer review only

of 100 mm) in our previous knee OA clinical trial with similar eligibility criteria. Using the control group data, we assume a between-participant SD of change in VAS pain of 24 mm. With 41 participants per arm, the study will have 80% power to detect a 15 mm difference in VAS pain between the intervention and control groups which is the minimum clinically important difference to be detected in OA trials[21], alpha 0.05, 2-sided significance. Based on a conservative 20% loss to follow up, we will recruit 102 participants (51 in each arm of the study).

Statistical analyses

Intention-to-treat analyses of primary and secondary outcomes will be presented, including all participants in their randomised groups. Comparisons between randomised groups of change in knee pain at 6 months will be analysed using ANCOVA, adjusting for baseline value for knee pain outcomes. Differences in knee pain trajectories over 6 months will be examined using linear mixed-effects models with baseline value as the covariate, fixed factors for treatment, time, and treatment x time interaction, and with an autoregressive AR (1) covariance matrix for repeated measures of individuals over time. Sensitivity analyses will be conducted for clinically important imbalances in baseline factors using multiple linear regression, or mixed models regression, as appropriate for the outcome measures. Multiple imputation of missing follow-up measures will be carried out as a sensitivity analysis when the percentage of missing data exceeds 5%. Subgroup analyses will be performed to examine whether the difference in outcomes between randomised groups varied based on sex, knee pain level and radiographic severity of knee OA. Analyses of treatment efficacy will be done by blinding individuals at the time of any protocol deviation and developing a model for the probability of deviation, followed by analyses using only the uncensored individuals where the weights are the inverse probability of censoring. This produces estimates of treatment effect as if there was full compliance with the protocol in this randomised controlled trial and is far preferable to per-protocol analyses based on (unweighted) observed compliance[28].

Data integrity and management

For peer review only

For peer review only - http://bmjopen.bmj.com/site/about/guidelines.xhtml

All data will be collected using Monash REDCap. Paper copies of questionnaires (if participants prefer to complete the questionnaires on hard copy) will be stored in locked filing cabinets, with restricted access. Electronic data will be stored in REDCap, and exported to a password-protected server after data collection, separating the identifying and non-identifying information. The codes linking data to identifying participant information will be kept separately from the study data, under password protection and with restricted access.

Due to the COVID-19, we will be providing a telehealth option for all clinic visits. This will be done in such a way that will not compromise participant safety or the scientific integrity of the trial. This study uses REDCap for consent and data collection, facilitating telehealth options. For participants who use the telehealth option for the screening/baseline visit, we will seek consent electronically (eConsent). REDCap has a feature that implements consent forms through an online survey which can be accessed on a computer, mobile phone, or tablet. The completed eConsent PDFs are stored in REDCap in a File Repository under "PDF Survey Archive".

Dissemination

Trial results, regardless of statistical significance, will be published in peer-reviewed journals and presented at national and international conferences. Upon publication of the primary manuscript, participants will be informed of their group allocation and provided with the results.

Discussion

This randomised controlled trial is conducted to determine whether metformin 2g daily over 6 months reduces knee pain in participants with symptomatic knee OA and concurrent overweight or obesity. If metformin proves effective in patients with symptomatic knee OA and concurrent overweight or obesity, it will offer an important therapeutic approach for obesity-metabolic syndrome phenotype of knee OA.

For peer review only

There are consistent chondroprotective, immunomodulatory and analgesic effects from metformin in pre-clinical cell and animal studies[13]. In pre-clinical studies, in addition to chondroprotective and anti-inflammatory effects, metformin was shown to be able to improve pain, such that rats or mice treated with metformin showed increased paw withdraw latency indicative of reduction in pain[13, 15, 16, 29]. In human studies, a randomised, double-blind trial showed that the combination of metformin with meloxicam improved knee pain by at least 50% more than meloxicam alone[30]. Additionally, one of the metformin's pleotropic effects is mild weight loss (~2.5%)[31], which is important when tackling the slow insidious weight creep from early to middle adulthood[32-34], particularly when obesity is a well-known risk factor for OA. Slowing weight gain over time not only has been proven to improve knee pain[35], but also was estimated to reduce knee replacement by up to 28.4%[36]. As such, metformin has the potential to play an important role in individuals who have knee OA with obesity-metabolic syndrome phenotype.

Studies have shown the beneficial effects of metformin in OA were mainly mediated by activation of the AMPK pathway[13]. As a key regulator of energy homeostasis and metabolism, activation of AMPK regulates key downstream enzymes involved in metabolism and transcription factors that regulate gene expression. As such, activation of the AMPK pathway in liver, muscle and adipose results in decreased lipogenesis and increased fatty acid oxidation, explaining some of the pleiotropic effects of metformin in improving metabolic profiles[10].

In summary, knee OA, specifically the obesity-metabolic syndrome phenotype, has limited effective treatment options. This study will provide high-quality evidence to determine whether metformin reduces knee pain in people with symptomatic knee OA with overweight or obesity over 6 months, with major clinical and public health importance for a potentially effective treatment option for knee OA to reduce knee pain and disease burden.

Acknowledgement

For peer review only - http://bmjopen.bmj.com/site/about/guidelines.xhtml

Ms Molly Bond, Mr Noor Abid, Mr Ashish Dinesh Nair, Dr Benjamin Sutu, Dr Talia Igel, Dr Luigi Zolio and Dr Rushab Shah have been involved in the coordination and/or execution of this study.

Authors' contributions

Conception and design: YW, DMU, AEW, FMC

Study execution and data acquisition: YZL, YW, MME, AEW, FMC

Drafting of the manuscript: YZL, YW, FMC

Critical revision of the manuscript for important intellectual content and approval of the final manuscript: YW, DMU, MME, AEW, SH, FMC

Obtaining of funding: FMC

Funding

The study is funded by an Investigator Grant from the National Health and Medical Research Council of Australia (NHMRC APP1194829).

YZL is the recipient of National Health and Medical Research Council (NHMRC) Clinical Postgraduate Scholarship (#1133903) and Royal Australasian College of Physicians Woolcock Scholarship. YW is the recipient of NHMRC Translating Research into Practice Fellowship (APP1168185). DMU is a recipient of a NHMRC/Medical Research Future Fund (MRFF) Career Development Fellowship (Clinical Level 2 #1142809). MME is the recipient of Bangabandhu Science and Technology Fellowship from Ministry of Science and Technology, Government of the People's Republic of Bangladesh. FMC is the recipient of NHMRC Investigator Grant (APP1194829). The funders of the study had no role in the study design and conduct of the study; collection, management, analysis and interpretation of the data; preparation, review, or approval of the manuscript; and decision to submit the manuscript for publication.

Competing interests

All authors declared no competing interests.

For peer review only

Availability of data and materials

Data sharing is not applicable to this article as no datasets were generated or analysed during the current study.

For peer review only

For peer review only - http://bmjopen.bmj.com/site/about/guidelines.xhtml

Figure 1. Flowchart of trial participation

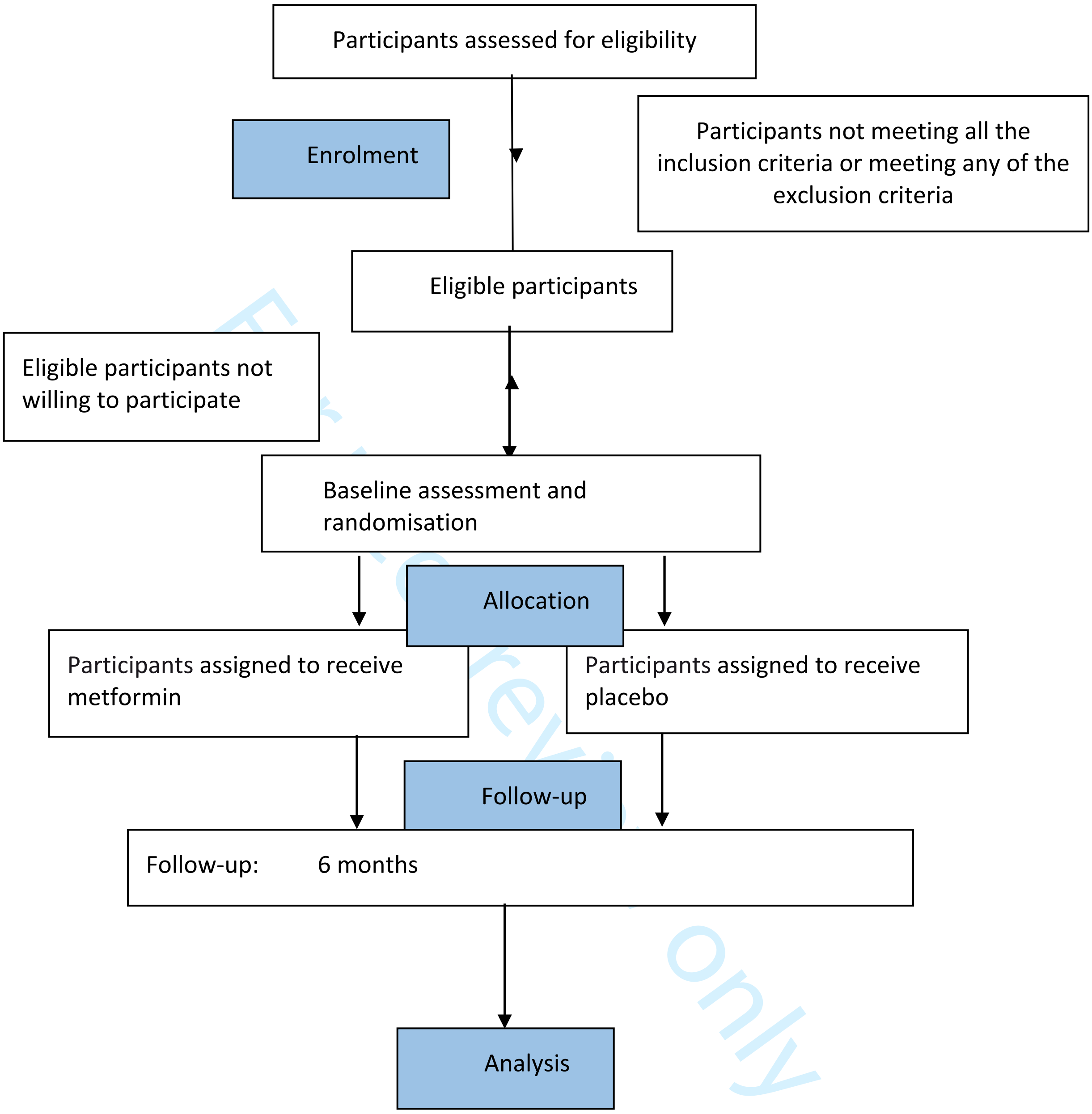

For peer review only - http://bmjopen.bmj.com/site/about/guidelines.xhtml

Table 1. Schedule of assessments

	Screening	Double-blind period		
	Screening / baseline assessment	Randomisation	1-5 months	6 months
**Study visit (onsite or telehealth)**	X			X
**Telephone interview (monthly)**			X	
**Informed consent**	X			
**Knee X-ray**	X			
**Blood test**	X			
**Medical history/conditions**	X			X
**Medication**	X			X
**Employment and education**	X			
**Smoking and alcohol**	X			
**Questionnaires**				
Knee VAS	X		X	X
WOMAC	X			X
PainDETECT	X			X
Hand VAS	X		X	X
Multi-site pain	X			X
AQoL	X			X
IPAQ	X			X
**Physical examination**				
Height, weight	X			X
**Compliance and safety (adverse events)**			X	X
**Dispense medication**		X		

For peer review only

AQoL: assessment of quality of life
IPAQ: international physical activity questionnaire
VAS: visual analogue scale
WOMAC: Western Ontario and McMaster Universities Osteoarthritis Index

References

1. Vos T, Lim SS, Abbafati C, Abbas KM, Abbasi M, Abbasifard M, Abbasi-Kangevari M, Abbastabar H, Abd-Allah F, Abdelalim A *et al*: Global burden of 369 diseases and injuries in 204 countries and territories, 1990–2019: a systematic analysis for the Global Burden of Disease Study 2019. *The Lancet* 2020, 396(10258):1204-1222.
2. Ferket BS, Feldman Z, Zhou J, Oei EH, Bierma-Zeinstra SMA, Mazumdar M: Impact of total knee replacement practice: cost effectiveness analysis of data from the Osteoarthritis Initiative. *BMJ* 2017, 356:j1131.
3. Ackerman IN, Bohensky MA, Zomer E, Tacey M, Gorelik A, Brand CA, de Steiger R: The projected burden of primary total knee and hip replacement for osteoarthritis in Australia to the year 2030. *BMC Musculoskelet Disord* 2019, 20(1):90.

4. van der Esch M, Knoop J, van der Leeden M, Roorda LD, Lems WF, Knol DL, Dekker J: Clinical phenotypes in patients with knee osteoarthritis: a study in the Amsterdam osteoarthritis cohort. *Osteoarthritis Cartilage* 2015, 23(4):544-549.
5. Knoop J, van der Leeden M, Thorstensson CA, Roorda LD, Lems WF, Knol DL, Steultjens MP, Dekker J: Identification of phenotypes with different clinical outcomes in knee osteoarthritis: Data from the osteoarthritis initiative. *Arthritis Care Res (Hoboken)* 2011, 63(11):1535-1542.
6. Thijssen E, van Caam A, van der Kraan PM: Obesity and osteoarthritis, more than just wear and tear: pivotal roles for inflamed adipose tissue and dyslipidaemia in obesity-induced osteoarthritis. *Rheumatology (Oxford, England)* 2015, 54(4):588-600.
7. Puenpatom RA, Victor TW: Increased prevalence of metabolic syndrome in individuals with osteoarthritis: an analysis of NHANES III data. *Postgrad Med* 2009, 121(6):9-20.
8. Berenbaum F, Eymard F, Houard X: Osteoarthritis, inflammation and obesity. *Curr Opin Rheumatol* 2013, 25(1):114-118.
9. Rojas LB, Gomes MB: Metformin: an old but still the best treatment for type 2 diabetes. *Diabetol Metab Syndr* 2013, 5(1):6.
10. Saisho Y: Metformin and Inflammation: Its Potential Beyond Glucose-lowering Effect. *Endocr Metab Immune Disord Drug Targets* 2015, 15(3):196-205.
11. Knowler WC, Fowler SE, Hamman RF, Christophi CA, Hoffman HJ, Brenneman AT, Brown-Friday JO, Goldberg R, Venditti E, Nathan DM: 10-year follow-up of diabetes incidence and weight loss in the Diabetes Prevention Program Outcomes Study. *Lancet* 2009, 374(9702):1677-1686.
12. Worsley R, Jane F, Robinson PJ, Bell RJ, Davis SR: Metformin for overweight women at midlife: a double-blind, randomized, controlled trial. *Climacteric* 2015, 18(2):270-277.
13. Lim YZ, Wang Y, Estee M, Abidi J, Udaya Kumar M, Hussain SM, Wluka AE, Little CB, Cicuttini FM: Metformin as a potential disease-modifying drug in osteoarthritis: a systematic review of pre-clinical and human studies. *Osteoarthritis Cartilage* 2022, 30(11):1434-1442.
14. Li H, Ding X, Terkeltaub R, Lin H, Zhang Y, Zhou B, He K, Li K, Liu Z, Wei J *et al*: Exploration of metformin as novel therapy for osteoarthritis: preventing cartilage degeneration and reducing pain behavior. *Arthritis Res Ther* 2020, 22(1):34.
15. Li J, Zhang B, Liu WX, Lu K, Pan H, Wang T, Oh CD, Yi D, Huang J, Zhao L *et al*: Metformin limits osteoarthritis development and progression through activation of AMPK signalling. *Ann Rheum Dis* 2020, 79(5):635-645.
16. Na HS, Kwon JY, Lee SY, Lee SH, Lee AR, Woo JS, Jung K, Cho KH, Choi JW, Lee DH *et al*: Metformin Attenuates Monosodium-Iodoacetate-Induced Osteoarthritis via Regulation of Pain Mediators and the Autophagy-Lysosomal Pathway. *Cells* 2021, 10(3).
17. Moher D, Hopewell S, Schulz KF, Montori V, Gøtzsche PC, Devereaux PJ, Elbourne D, Egger M, Altman DG: CONSORT 2010 Explanation and Elaboration: updated guidelines for reporting parallel group randomised trials. *BMJ* 2010, 340:c869.
18. Altman R, Asch E, Bloch D, Bole G, Borenstein D, Brandt K, Christy W, Cooke TD, Greenwald R, Hochberg M *et al*: Development of criteria for the classification and reporting of osteoarthritis. Classification of osteoarthritis of the knee. Diagnostic and Therapeutic Criteria Committee of the American Rheumatism Association. *Arthritis and rheumatism* 1986, 29(8):1039-1049.
19. Australian Public Assessment Report for Linagliptin / Metformin HCl. Proprietary Product Name: Trajentamet. Therapeutics Goods Administration Health Safety Regulation. September 2013. In.
20. Pham T, van der Heijde D, Altman RD, Anderson JJ, Bellamy N, Hochberg M, Simon L, Strand V, Woodworth T, Dougados M: OMERACT-OARSI initiative: Osteoarthritis Research Society International set of responder criteria for osteoarthritis clinical trials revisited. *Osteoarthritis Cartilage* 2004, 12(5):389-399.

For peer review only - http://bmjopen.bmj.com/site/about/guidelines.xhtml

21. Tubach F, Ravaud P, Baron G, Falissard B, Logeart I, Bellamy N, Bombardier C, Felson D, Hochberg M, van der Heijde D *et al*: Evaluation of clinically relevant changes in patient reported outcomes in knee and hip osteoarthritis: the minimal clinically important improvement. *Annals of the rheumatic diseases* 2005, 64(1):29-33.
22. Bellamy N, Buchanan WW, Goldsmith CH, Campbell J, Stitt LW: Validation study of WOMAC: a health status instrument for measuring clinically important patient relevant outcomes to antirheumatic drug therapy in patients with osteoarthritis of the hip or knee. *J Rheumatol* 1988, 15(12):1833-1840.
23. Osborne RH, Hawthorne G, Lew EA, Gray LC: Quality of life assessment in the community-dwelling elderly: validation of the Assessment of Quality of Life (AQoL) Instrument and comparison with the SF-36. *J Clin Epidemiol* 2003, 56(2):138-147.
24. Hochman JR, Gagliese L, Davis AM, Hawker GA: Neuropathic pain symptoms in a community knee OA cohort. *Osteoarthritis Cartilage* 2011, 19(6):647-654.
25. Craig CL, Marshall AL, Sjöström M, Bauman AE, Booth ML, Ainsworth BE, Pratt M, Ekelund U, Yngve A, Sallis JF *et al*: International physical activity questionnaire: 12-country reliability and validity. *Med Sci Sports Exerc* 2003, 35(8):1381-1395.
26. Kloppenburg M, Maheu E, Kraus VB, Cicuttini F, Doherty M, Dreiser RL, Henrotin Y, Jiang GL, Mandl L, Martel-Pelletier J *et al*: OARSI Clinical Trials Recommendations: Design and conduct of clinical trials for hand osteoarthritis. *Osteoarthritis Cartilage* 2015, 23(5):772-786.
27. Wluka AE, Ding C, Jones G, Cicuttini FM: The clinical correlates of articular cartilage defects in symptomatic knee osteoarthritis: a prospective study. *Rheumatology (Oxford)* 2005, 44(10):1311-1316.
28. Hernán MA, Lanoy E, Costagliola D, Robins JM: Comparison of dynamic treatment regimes via inverse probability weighting. *Basic Clin Pharmacol Toxicol* 2006, 98(3):237-242.
29. Park MJ, Moon SJ, Baek JA, Lee EJ, Jung KA, Kim EK, Kim DS, Lee JH, Kwok SK, Min JK *et al*: Metformin Augments Anti-Inflammatory and Chondroprotective Properties of Mesenchymal Stem Cells in Experimental Osteoarthritis. *J Immunol* 2019, 203(1):127-136.
30. Mohammed M, Al-Shamma K, Jassim N: Evaluation of the Clinical use of Metformin or Pioglitazone in Combination with Meloxicam in Patients with Knee Osteoarthritis; using Knee Injury and Osteoarthritis outcome Score. *Iraqi Journal of Pharmaceutical Sciences* 2014, 23(2):13-23.
31. Shi Q, Wang Y, Hao Q, Vandvik PO, Guyatt G, Li J, Chen Z, Xu S, Shen Y, Ge L *et al*: Pharmacotherapy for adults with overweight and obesity: a systematic review and network meta-analysis of randomised controlled trials. *Lancet* 2022, 399(10321):259-269.
32. Brebal KMM, Silveira J, Menezes RCE, Epifânio SBO, Marinho PM, Longo-Silva G: Weight gain and changes in nutritional status of Brazilian adults after 20 years of age: a time-trend analysis (2006-2012). *Rev Bras Epidemiol* 2020, 23:e200045.
33. Peeters A, Magliano DJ, Backholer K, Zimmet P, Shaw JE: Changes in the rates of weight and waist circumference gain in Australian adults over time: a longitudinal cohort study. *BMJ Open* 2014, 4(1):e003667.
34. Zheng Y, Manson JE, Yuan C, Liang MH, Grodstein F, Stampfer MJ, Willett WC, Hu FB: Associations of Weight Gain From Early to Middle Adulthood With Major Health Outcomes Later in Life. *JAMA* 2017, 318(3):255-269.
35. Wang Y, Lombard C, Hussain SM, Harrison C, Kozica S, Brady SRE, Teede H, Cicuttini FM: Effect of a low-intensity, self-management lifestyle intervention on knee pain in community-based young to middle-aged rural women: a cluster randomised controlled trial. *Arthritis research & therapy* 2018, 20(1):74.
36. Hussain SM, Ackerman IN, Wang Y, English DR, Wluka AE, Giles GG, Cicuttini FM: Trajectories of body mass index from early adulthood to late midlife and incidence of total knee arthroplasty for osteoarthritis: findings from a prospective cohort study. *Osteoarthritis Cartilage* 2023, 31(3):397-405.

For peer review only - http://bmjopen.bmj.com/site/about/guidelines.xhtml

SPIRIT

STANDARD PROTOCOL ITEMS: RECOMMENDATIONS FOR INTERVENTIONAL TRIALS

SPIRIT 2013 Checklist: Recommended items to address in a clinical trial protocol and related documents*

Section/item	Item No	Description	Page
**Administrative information**			
Title	1	Descriptive title identifying the study design, population, interventions, and, if applicable, trial acronym	**1**
Trial registration	2a	Trial identifier and registry name. If not yet registered, name of intended registry	2, 5
	2b	All items from the World Health Organization Trial Registration Data Set	NA
Protocol version	3	Date and version identifier	NA
Funding	4	Sources and types of financial, material, and other support	1
Roles and responsibilities	5a	Names, affiliations, and roles of protocol contributors	1, 13
	5b	Name and contact information for the trial sponsor	1, 13
	5c	Role of study sponsor and funders, if any, in study design; collection, management, analysis, and interpretation of data; writing of the report; and the decision to submit the report for publication, including whether they will have ultimate authority over any of these activities	1, 13
	5d	Composition, roles, and responsibilities of the coordinating centre, steering committee, endpoint adjudication committee, data management team, and other individuals or groups overseeing the trial, if applicable (see Item 21a for data monitoring committee)	NA
**Introduction**			
Background and rationale	6a	Description of research question and justification for undertaking the trial, including summary of relevant studies (published and unpublished) examining benefits and harms for each intervention	4
	6b	Explanation for choice of comparators	4
Objectives	7	Specific objectives or hypotheses	4,5
Trial design	8	Description of trial design including type of trial (eg, parallel group, crossover, factorial, single group), allocation ratio, and framework (eg, superiority, equivalence, noninferiority, exploratory)	4

For peer review only

For peer review only - http://bmjopen.bmj.com/site/about/guidelines.xhtml

Methods: Participants, interventions, and outcomes

Study setting	9	Description of study settings (eg, community clinic, academic hospital) and list of countries where data will be collected. Reference to where list of study sites can be obtained	5
Eligibility criteria	10	Inclusion and exclusion criteria for participants. If applicable, eligibility criteria for study centres and individuals who will perform the interventions (eg, surgeons, psychotherapists)	5,6
Interventions	11a	Interventions for each group with sufficient detail to allow replication, including how and when they will be administered	7
	11b	Criteria for discontinuing or modifying allocated interventions for a given trial participant (eg, drug dose change in response to harms, participant request, or improving/worsening disease)	7
	11c	Strategies to improve adherence to intervention protocols, and any procedures for monitoring adherence (eg, drug tablet return, laboratory tests)	7
	11d	Relevant concomitant care and interventions that are permitted or prohibited during the trial	7
Outcomes	12	Primary, secondary, and other outcomes, including the specific measurement variable (eg, systolic blood pressure), analysis metric (eg, change from baseline, final value, time to event), method of aggregation (eg, median, proportion), and time point for each outcome. Explanation of the clinical relevance of chosen efficacy and harm outcomes is strongly recommended	8
Participant timeline	13	Time schedule of enrolment, interventions (including any run-ins and washouts), assessments, and visits for participants. A schematic diagram is highly recommended (see Figure)	16
Sample size	14	Estimated number of participants needed to achieve study objectives and how it was determined, including clinical and statistical assumptions supporting any sample size calculations	9, 10
Recruitment	15	Strategies for achieving adequate participant enrolment to reach target sample size	5

Methods: Assignment of interventions (for controlled trials)

Allocation:

Sequence generation	16a	Method of generating the allocation sequence (eg, computer-generated random numbers), and list of any factors for stratification. To reduce predictability of a random sequence, details of any planned restriction (eg, blocking) should be provided in a separate document that is unavailable to those who enrol participants or assign interventions	6

Allocation concealment mechanism	16b	Mechanism of implementing the allocation sequence (eg, central telephone; sequentially numbered, opaque, sealed envelopes), describing any steps to conceal the sequence until interventions are assigned	6
Implementation	16c	Who will generate the allocation sequence, who will enrol participants, and who will assign participants to interventions	6
Blinding (masking)	17a	Who will be blinded after assignment to interventions (eg, trial participants, care providers, outcome assessors, data analysts), and how	6
	17b	If blinded, circumstances under which unblinding is permissible, and procedure for revealing a participant's allocated intervention during the trial	6,7
**Methods: Data collection, management, and analysis**			
Data collection methods	18a	Plans for assessment and collection of outcome, baseline, and other trial data, including any related processes to promote data quality (eg, duplicate measurements, training of assessors) and a description of study instruments (eg, questionnaires, laboratory tests) along with their reliability and validity, if known. Reference to where data collection forms can be found, if not in the protocol	7,8
	18b	Plans to promote participant retention and complete follow-up, including list of any outcome data to be collected for participants who discontinue or deviate from intervention protocols	8
Data management	19	Plans for data entry, coding, security, and storage, including any related processes to promote data quality (eg, double data entry; range checks for data values). Reference to where details of data management procedures can be found, if not in the protocol	10, 11
Statistical methods	20a	Statistical methods for analysing primary and secondary outcomes. Reference to where other details of the statistical analysis plan can be found, if not in the protocol	10
	20b	Methods for any additional analyses (eg, subgroup and adjusted analyses)	10
	20c	Definition of analysis population relating to protocol non-adherence (eg, as randomised analysis), and any statistical methods to handle missing data (eg, multiple imputation)	10
**Methods: Monitoring**			
Data monitoring	21a	Composition of data monitoring committee (DMC); summary of its role and reporting structure; statement of whether it is independent from the sponsor and competing interests; and reference to where further details about its charter can be found, if not in the protocol. Alternatively, an explanation of why a DMC is not needed	7

	21b	Description of any interim analyses and stopping guidelines, including who will have access to these interim results and make the final decision to terminate the trial	7
Harms	22	Plans for collecting, assessing, reporting, and managing solicited and spontaneously reported adverse events and other unintended effects of trial interventions or trial conduct	7
Auditing	23	Frequency and procedures for auditing trial conduct, if any, and whether the process will be independent from investigators and the sponsor	NA
**Ethics and dissemination**			
Research ethics approval	24	Plans for seeking research ethics committee/institutional review board (REC/IRB) approval	5
Protocol amendments	25	Plans for communicating important protocol modifications (eg, changes to eligibility criteria, outcomes, analyses) to relevant parties (eg, investigators, REC/IRBs, trial participants, trial registries, journals, regulators)	5
Consent or assent	26a	Who will obtain informed consent or assent from potential trial participants or authorised surrogates, and how (see Item 32)	5
	26b	Additional consent provisions for collection and use of participant data and biological specimens in ancillary studies, if applicable	NA
Confidentiality	27	How personal information about potential and enrolled participants will be collected, shared, and maintained in order to protect confidentiality before, during, and after the trial	11
Declaration of interests	28	Financial and other competing interests for principal investigators for the overall trial and each study site	13
Access to data	29	Statement of who will have access to the final trial dataset, and disclosure of contractual agreements that limit such access for investigators	13
Ancillary and post-trial care	30	Provisions, if any, for ancillary and post-trial care, and for compensation to those who suffer harm from trial participation	7
Dissemination policy	31a	Plans for investigators and sponsor to communicate trial results to participants, healthcare professionals, the public, and other relevant groups (eg, via publication, reporting in results databases, or other data sharing arrangements), including any publication restrictions	11
	31b	Authorship eligibility guidelines and any intended use of professional writers	13
	31c	Plans, if any, for granting public access to the full protocol, participant-level dataset, and statistical code	14

For peer review only - http://bmjopen.bmj.com/site/about/guidelines.xhtml

Appendices

Informed consent materials	32	Model consent form and other related documentation given to participants and authorised surrogates	https://www.monash.edu/medicine/sphpm/units/musculoskeletal-epidemiology/join-our-studies
Biological specimens	33	Plans for collection, laboratory evaluation, and storage of biological specimens for genetic or molecular analysis in the current trial and for future use in ancillary studies, if applicable	NA

*It is strongly recommended that this checklist be read in conjunction with the SPIRIT 2013 Explanation & Elaboration for important clarification on the items. Amendments to the protocol should be tracked and dated. The SPIRIT checklist is copyrighted by the SPIRIT Group under the Creative Commons "Attribution-NonCommercial-NoDerivs 3.0 Unported" license.

For peer review only

BMJ Open

Metformin for knee osteoarthritis with obesity: study protocol for a randomised, double-blind, placebo-controlled trial

Journal:	*BMJ Open*
Manuscript ID	bmjopen-2023-079489.R1
Article Type:	Protocol
Date Submitted by the Author:	24-Nov-2023

Complete List of Authors:	Lim, Yuan ; Monash University School of Public Health and Preventive Medicine<br>Wang, Yuanyuan; Monash University School of Public Health and Preventive Medicine<br>Urquhart, Donna M.; Monash University School of Public Health and Preventive Medicine<br>Estee, Mahnuma; Monash University School of Public Health and Preventive Medicine<br>Wluka, Anita; Monash University School of Public Health and Preventive Medicine<br>Heritier, Stephane; Monash University School of Public Health and Preventive Medicine<br>Cicuttini, Flavia; Monash University School of Public Health and Preventive Medicine

**Primary Subject Heading**:	Rheumatology
Secondary Subject Heading:	Rheumatology, Pharmacology and therapeutics
Keywords:	Obesity, Knee < ORTHOPAEDIC & TRAUMA SURGERY, Randomized Controlled Trial

SCHOLARONE™ Manuscripts

Metformin for knee osteoarthritis with obesity: study protocol for a randomised, double-blind, placebo-controlled trial

Yuan Z Lim[1]*, Yuanyuan Wang[1]*, Donna M Urquhart[1], Mahnuma Mahfuz Estee[1], Anita E. Wluka[1], Stephane Heritier[1], Flavia M. Cicuttini[1]

[1]School of Public Health and Preventive Medicine, Monash University, Melbourne, VIC 3004, Australia.

*YZL and YW contributed equally

Correspondence to:

Professor Flavia Cicuttini, School of Public Health and Preventive Medicine, Monash University, 553 St Kilda Road, Melbourne, VIC 3004, Australia

Telephone: +61 3 9903 0158

Email: flavia.cicuttini@monash.edu

For peer review only

Abstract

Introduction

Over half of the populations with knee osteoarthritis (OA) have obesity. These individuals have many other shared metabolic risk factors. Metformin is a safe, inexpensive, well-tolerated drug that has pleotropic effects, including structural protection, anti-inflammatory and analgesic effects in OA, specifically the knee. The aim of this randomised, double-blind, placebo-controlled trial is to determine whether metformin reduces knee pain over 6 months in individuals with symptomatic knee OA who are overweight or obese.

Methods and analysis

One hundred and two participants with symptomatic knee OA and overweight or obesity will be recruited from the community in Melbourne, Australia, and randomly allocated in a 1:1 ratio to receive either metformin 2g or identical placebo daily for 6 months. The primary outcome is reduction of knee pain (assessed by 100mm visual analogue scale) at 6 months. The secondary outcomes are OMERACT-OARSI responder criteria [Western Ontario and McMaster Universities Osteoarthritis Index (WOMAC) pain, function and participant's global assessment (VAS)] at 6 months; change in knee pain, stiffness, function using WOMAC at 6 months and quality of life at 6 months. Adverse events will be recorded. The primary analysis will be by intention to treat, including all participants in their randomised groups.

Ethics and dissemination

Ethics approval has been obtained from the Alfred Hospital Ethics Committee (708/20) and Monash University Human Research Ethics Committee (28498). Written informed consent will be obtained from all the participants. The findings will be disseminated through peer-review publications and conference presentations.

Trial registration

Australian New Zealand Clinical Trials Registry, ACTRN12621000710820 (registered 8th June, 2021).

For peer review only - http://bmjopen.bmj.com/site/about/guidelines.xhtml

For peer review only

STRENGTHS AND LIMITATIONS OF THIS STUDY

- This study is a randomised, double-blind, placebo-controlled trial.
- This study will provide high-quality evidence to address whether metformin has an analgesic effect over 6 months in individuals with symptomatic knee OA with overweight or obesity.
- The generalisability of this study result will be limited to those without diabetes or those not requiring anti-hyperglycaemic therapy.

Keywords

Metformin, knee osteoarthritis, obesity, pain

For peer review only

For peer review only - http://bmjopen.bmj.com/site/about/guidelines.xhtml

INTRODUCTION

Osteoarthritis (OA) is a leading cause of global disability, resulting in 19 million years lived with disability in 2019[1]. There is no approved disease-modifying treatment for OA to date. With limited effective therapies, end-stage OA is treated with total joint replacement, estimated to cost about $10 billion/year in the United States[2] and over $AUD 1 billion/year in Australia[3]. Despite being a multifactorial disease, management of OA has taken a 'one size fits all' approach without considering the different pathological pathways and OA phenotypes, resulting in poor patient outcomes. One distinctive knee OA phenotype is the obese phenotype[4, 5], mediated by inflammatory and metabolic mechanisms[6]. Over 50% of knee OA patients have obesity[7]. Given obesity and obesity-related metabolic factors (hyperglycaemia, dyslipidaemia, hypertension) are all risk factors for knee OA[6, 8], drugs targeting obesity and its associated inflammatory and metabolic abnormalities have the potential to affect the pathogenesis of knee OA.

Metformin is a safe, inexpensive, well-tolerated oral biguanide, which is not only widely used for treatment of type 2 diabetes for over 60 years, but also has a long history of safe use in non-diabetic populations[9, 10]. Additional to its glucose lowering effects, metformin modulates metabolic factors, resulting in at least 2-3kg of weight loss[11, 12] and reduced inflammation and plasma lipids[9, 10]. A recent systematic review of animal and human studies showed metformin has structural protective, anti-inflammatory and analgesic effect for OA, specifically for the knee[13]. These pleotropic effects of metformin are mainly driven by the activation of AMP-activated protein kinase (AMPK) pathway[14-16]. Hence, metformin has the potential to reduce pain in those with knee OA and overweight or obesity. This study aims to determine the effect of metformin on reducing knee pain in people with symptomatic knee OA and overweight or obesity.

METHODS AND ANALYSIS

Study design

For peer review only - http://bmjopen.bmj.com/site/about/guidelines.xhtml

This is a randomised, double-blind, placebo-controlled trial in people with symptomatic knee OA and overweight or obesity, to determine the effect of metformin 2g daily versus placebo on reducing knee pain over 6 months.

Hypothesis and objectives

It is hypothesised that metformin, compared with placebo, will (1) reduce knee pain (primary hypothesis); (2) improve clinical outcomes (stiffness, function, and health-related quality of life); and that (3) the effect of metformin on knee pain and function will be associated with changes in inflammatory and metabolic biomarkers and/or weight loss. If metformin is proven to be effective, it will provide a safe, low-cost treatment to reduce pain and improve function for people with symptomatic knee OA with concurrent overweight or obesity.

Trial registration and reporting

The trial was registered at the Australian New Zealand Clinical Trials Registry prior to commencing recruitment (ACTRN12621000710820, registered 8th June, 2021). The trial reporting will be guided by the Consolidated Standards of Reporting Trials (CONSORT) Statement[17].

Study setting and participants

Participants with symptomatic knee OA and overweight or obesity will be recruited using a combined strategy including collaboration with medical practitioners and advertisements in social and local media. This single centre study will be conducted in Melbourne, Australia.

Inclusion criteria

(1) Men and women aged >40 years, with overweight or obesity (body mass index ≥25 kg/m^2); (2) Knee pain for at least 6 months with a pain score ≥40 mm on a 100 mm visual analogue scale (VAS); (3) Meet the American College of Rheumatology clinical criteria for knee OA[18].

Exclusion criteria

For peer review only - http://bmjopen.bmj.com/site/about/guidelines.xhtml

(1) Severe radiographic knee OA (Kellgren-Lawrence grade 4) or severe knee pain (on standing >80 mm on a 100 mm VAS); (2) Any inflammatory arthritis including rheumatoid arthritis, psoriatic arthritis, crystal arthritis, spondyloarthritis, connective-tissue disease associated arthritis or reactive arthritis or significant knee injury; (3) Known or newly diagnosed diabetes requiring anti-hyperglycemic therapy or previous adverse reaction to metformin; (4) Index knee surgery (arthroscopy or open surgery) in the past year; (5) Index knee intra-articular hyaluronic acid injection in the past 6 months or corticosteroid injection in the past 3 months; (6) Use of any investigational drugs or device within 30 days prior to randomisation; (7) Index knee planned joint replacement or arthroscopy in the next 6 months; (8) Other muscular, joint or neurological condition affecting lower limb function; (9) Acute or chronic renal or liver impairment; (10) Other medical condition precluding study participation or relocation; (11) Women who are pregnant, lactating or trying to become pregnant. Use of menopausal hormone therapy or oral contraceptive pill will be permitted so long as the dose has been stable for at least 30 days prior to study entry.

Study timeline

This trial began recruitment on 16th June, 2021. It is estimated to finish recruitment on 30th September, 2023 and complete the 6-month follow-up and data collection in March, 2024. Figure 1 shows trial participation and study procedure.

Randomisation, allocation concealment and blinding

Allocation of participants in a 1:1 ratio to one of the two groups will be by computer generated random numbers prepared by a statistician with no involvement in the trial. Block randomisation using random permuted blocks of sizes 4 and 6 will be performed. The use of a central automated allocation procedure with security in place will ensure the allocation cannot be accessed or influenced by any person. Allocation concealment and double blinding will be ensured by: (1) medications being dispensed by Syntro Pharmacy Pty Ltd; (2) use of an identical placebo tablet; (3) subjective measures being taken by research assistants blinded to group allocation. Participants, assessors and statisticians will be blinded to group allocation.

Intervention and dosing

For peer review only - http://bmjopen.bmj.com/site/about/guidelines.xhtml

For peer review only

All participants will undergo usual care by their treating health practitioners. Eligible participants will be randomly assigned to receive either metformin (up to 2000 mg) once daily or placebo once daily. Treatment with study drug will be initiated at a dose of 500 mg once a day with the evening meal. Over 6 weeks, the dose of study drug will be titrated to 2000 mg once daily (or placebo once daily) to minimise gastrointestinal side effects.

Safety

Any adverse events or serious adverse event will be reported during the study. Blood tests will occur at screening. No data safety monitoring board is required as this agent is approved by Therapeutic Goods Administration with well-known safety profile[19]. Unblinding participants due to side effects of metformin was not an issue in a previous clinical trial[12].

Compliance

Compliance with trial medication will be assessed at 6 months by pill count. Study staff will phone participants in the middle between study visits to monitor medication adherence. Monthly telephone contact for the first 5 months will be conducted to address any concerns, as well as following up knee pain outcome (VAS). This will help to mitigate non-compliance.

Concomitant medication

To maintain the pragmatic nature of the trial, there are no restrictions with regards to concomitant medications, including glucosamine, chondroitin, non-steroidal anti-inflammatory drugs, and opioids analgesics, which will be allowed during the trial and be recorded by questionnaire at all visits. Patients will be asked to keep medications as stable as possible and use paracetamol as rescue medication.

Study procedures

Participants will be screened via phone by questionnaire before attending the screening visit via telehealth. There will be 2 study visits (onsite or telehealth): screening/baseline and month 6, as shown in Table 1. At screening, participants will complete questionnaires, have a knee X-ray and blood tests

For peer review only - http://bmjopen.bmj.com/site/about/guidelines.xhtml

[renal and liver function, plasma glucose and lipids, vitamin B12, and inflammatory biomarkers (C-reactive protein, tumour necrosis factor, interleukin-1 and interleukin 6)] to ensure inclusion criteria are met and exclusion criteria are absent. The index knee will be defined as having symptomatic OA. If both knees are symptomatic and eligible based on VAS pain, the one with higher VAS pain score will be used; if both knees are symptomatic with the same pain level, the one with least severe radiographic OA (joint space narrowing) will be used; if both knees have the same pain level and radiographic severity of OA, the dominant knee will be used. Physical examinations and questionnaires will be performed at months 0 and 6. The same researchers, blinded to treatment allocation, will measure all clinical variables, administer questionnaires, monitor compliance, and record adverse events. Participants are able to withdraw at any time during the trial; the time and reasons will be recorded. If participants withdraw from the study, they will be requested to complete questionnaires (posted to the participants with return envelope).

Table 1. Schedule of assessments

	**Screening**	**Double-blind period**		
	**Screening/ baseline assessment**	**Random isation**	**1-5 months**	**6 months**
**Study visit (onsite or telehealth)**	X			X
**Telephone interview (monthly)**			X	
**Informed consent**	X			
**Knee X-ray**	X			
**Blood test**	X			
**Medical history/conditions**	X			X
**Medication**	X			X
**Employment and education**	X			
**Smoking and alcohol**	X			
**Questionnaires**				
Knee VAS	X		X	X
WOMAC	X			X
PainDETECT	X			X
Hand VAS	X		X	X
Multi-site pain	X			X
AQoL	X			X
IPAQ	X			X
**Physical examination**				
Height, weight*	X			X
**Compliance and safety (adverse**			X	X

For peer review only - http://bmjopen.bmj.com/site/about/guidelines.xhtml

**events)**				
**Dispense medication**		X		

*Height and weight will be self-reported if the visit is via telehealth.
AQoL: assessment of quality of life.
IPAQ: international physical activity questionnaire.
VAS: visual analogue scale.
WOMAC: Western Ontario and McMaster Universities Osteoarthritis Index.

Primary outcome

The primary outcome is pain reduction at 6 months, measured by change in VAS knee pain from baseline to 6 months (follow-up VAS pain score - baseline score). Knee pain will be measured at baseline and monthly follow-up using a 100 mm VAS by asking "on this line, where would you rate your knee pain over the last 7 days?" with terminal descriptors 'no pain' (score 0) and 'worst imaginable pain' (score 100).

For peer review only

Secondary outcomes

OMERACT-OARSI responder criteria

This will be used to define a responder based on improvement in WOMAC pain and function and the participant's global assessment[20] at 6 months. Participant's global assessment will be evaluated by 100-mm VAS[21].

Change in knee pain, stiffness and function

Knee pain, stiffness and function will be assessed using the Western Ontario and McMaster Universities Osteoarthritis Index (WOMAC)[22] at baseline and 6 months.

Health-related quality of life

This will be measured using the Assessment of Quality of Life (AQoL)[23] at baseline and 6 months.

Other measures

Descriptive data: Data regarding age, gender, height, weight, duration of symptoms, employment, medical history, medication use, education level, smoking, alcohol consumption will be collected using a questionnaire at baseline.

For peer review only - http://bmjopen.bmj.com/site/about/guidelines.xhtml

PainDETECT: PainDETECT is a validated questionnaire used to assess pain sensitisation in OA[24], and will be assessed at baseline and 6 months.

Physical activity: Physical activity will be measured using the International Physical Activity Questionnaire (IPAQ)[25] short version at baseline and 6 months.

Hand VAS: Pain reduction in hands will be measured at baseline, then monthly for 6 months, according to the OARSI recommendations for the design and conduct of clinical trials for hand OA, which recommend the use of single question pain VAS[26].

Multi-site pain: The presence and levels of multi-site musculoskeletal pain will be assessed at baseline and 6 months using a questionnaire.

Adverse events: These will be measured in a log-book by the blinded assessor at each follow-up.

Biochemical parameters: General (cell counts, liver and renal function), inflammatory biomarkers (C-reactive protein, erythrocyte sedimentation rate, interleukin-6, tumour necrosis factor), plasma glucose and lipids, and vitamin B12 will be measured at baseline and 6 months.

Knee X-ray

X-ray of the study knee (weight-bearing anteroposterior view) will be scored using Kellgren-Lawrence grade. Our intra- and inter-observer reliability is 0.93 and 0.86 for osteophytes, 0.93 and 0.85 for joint space narrowing, respectively[27].

Sample size calculation

The primary outcome is change in VAS knee pain over 6 months. The mean VAS pain was 55 mm (out of 100 mm) in our previous knee OA clinical trial with similar eligibility criteria[28, 29]. Using the control group data, we assume a between-participant SD of change in VAS pain of 24 mm. With 41 participants per arm, the study will have 80% power to detect a 15 mm difference in VAS pain between the intervention and control groups which is the minimum clinically important difference to be detected in OA trials[21], alpha 0.05, 2-sided significance. Based on our previous knee OA trials [28, 30-32], with a conservative assumption of 20% loss to follow up, we will recruit 102 participants (51 in each arm of the study).

Statistical analyses

For peer review only - http://bmjopen.bmj.com/site/about/guidelines.xhtml

Intention-to-treat analyses of primary and secondary outcomes will be presented, including all participants in their randomised groups. Comparisons between randomised groups of change in knee pain at 6 months will be analysed using ANCOVA, adjusting for baseline value for knee pain outcomes. Differences in knee pain trajectories over 6 months will be examined using linear mixed-effects models with baseline value as the covariate, fixed factors for treatment, time, and treatment x time interaction, and with an autoregressive AR (1) covariance matrix for repeated measures of individuals over time. Sensitivity analyses will be conducted for clinically important imbalances in baseline factors using multiple linear regression, or mixed models regression, as appropriate for the outcome measures. Multiple imputation of missing follow-up measures will be carried out as a sensitivity analysis when the percentage of missing data exceeds 5%. Subgroup analyses will be performed to examine whether the difference in outcomes between randomised groups varied based on sex, knee pain level and radiographic severity of knee OA. Analyses of treatment efficacy will be done by blinding individuals at the time of any protocol deviation and developing a model for the probability of deviation, followed by analyses using only the uncensored individuals where the weights are the inverse probability of censoring. This produces estimates of treatment effect as if there was full compliance with the protocol in this randomised controlled trial and is far preferable to per-protocol analyses based on (unweighted) observed compliance[33].

Data integrity and management

All data will be collected using Monash REDCap. Paper copies of questionnaires (if participants prefer to complete the questionnaires on hard copy) will be stored in locked filing cabinets, with restricted access. Electronic data will be stored in REDCap, and exported to a password-protected server after data collection, separating the identifying and non-identifying information. The codes linking data to identifying participant information will be kept separately from the study data, under password protection and with restricted access.

Due to the COVID-19, we will be providing a telehealth option for all clinic visits. This will be done in such a way that will not compromise participant safety or the scientific integrity of the trial. This study

For peer review only - http://bmjopen.bmj.com/site/about/guidelines.xhtml

uses REDCap for consent and data collection, facilitating telehealth options. For participants who use the telehealth option for the screening/baseline visit, we will seek consent electronically (eConsent). REDCap has a feature that implements consent forms through an online survey which can be accessed on a computer, mobile phone, or tablet. The completed eConsent PDFs are stored in REDCap in a File Repository under "PDF Survey Archive". Physical examination will not be possible with telehealth option. Thus, height and weight will be self-reported.

Patient and public involvement

This study was informed by identification of clinical need in patients with OA attending our clinics. The clinical need and approach to translation is informed by the work with Musculoskeletal Australia in systematic reviews of consumers' needs in OA[34]. Once the trial has been published, participants will receive a study newsletter with details of the results which is suitable for a non-specialist audience.

ETHICS AND DISSEMINATION

Ethics approval has been obtained from the Alfred Hospital Ethics Committee (708/20) and Monash University Human Research Ethics Committee (28498). Written informed consent will be obtained from all the participants (Supplemental document). Trial results, regardless of statistical significance, will be published in peer-reviewed journals and presented at national and international conferences. Upon publication of the primary manuscript, participants will be informed of their group allocation and provided with the results.

DISCUSSION

This randomised controlled trial is conducted to determine whether metformin 2g daily over 6 months reduces knee pain in participants with symptomatic knee OA and concurrent overweight or obesity. If metformin proves effective in patients with symptomatic knee OA and concurrent overweight or obesity, it will offer an important therapeutic approach for obesity-metabolic syndrome phenotype of knee OA.

For peer review only - http://bmjopen.bmj.com/site/about/guidelines.xhtml

There are consistent chondroprotective, immunomodulatory and analgesic effects from metformin in pre-clinical cell and animal studies[13]. In pre-clinical studies, in addition to chondroprotective and anti-inflammatory effects, metformin was shown to be able to improve pain, such that rats or mice treated with metformin showed increased paw withdraw latency indicative of reduction in pain[13, 15, 16, 35]. In human studies, a randomised, double-blind trial showed that the combination of metformin with meloxicam improved knee pain by at least 50% more than meloxicam alone[36]. Additionally, one of the metformin's pleotropic effects is mild weight loss (~2.5%)[37], which is important when tackling the slow insidious weight creep from early to middle adulthood[38-40], particularly when obesity is a well-known risk factor for OA, and for more symptomatic and more progressive knee OA. Slowing weight gain over time not only has been proven to improve knee pain[41], but also was estimated to reduce knee replacement by up to 28.4%[42]. As such, metformin has the potential to play an important role in individuals who have knee OA with obesity-metabolic syndrome phenotype.

Studies have shown the beneficial effects of metformin in OA were mainly mediated by activation of the AMPK pathway[13]. As a key regulator of energy homeostasis and metabolism, activation of AMPK regulates key downstream enzymes involved in metabolism and transcription factors that regulate gene expression. As such, activation of the AMPK pathway in liver, muscle and adipose results in decreased lipogenesis and increased fatty acid oxidation, explaining some of the pleiotropic effects of metformin in improving metabolic profiles[10].

The study has several strengths. It is a randomised, double-blind, placebo-controlled trial which will provide high-quality evidence to address the aim of this study. Nevertheless, our study population is limited to those without a valid indication for metformin use, as it would be unethical to withhold metformin with a clinical indication, specifically people with diabetes, thus limiting the generalisability of the study results. The diabetic population is known to have more obesity and concurrent metabolic syndrome[43], and it is likely that those with diabetes and knee OA who will be excluded from this study, are the populations at greatest need for metformin, which may underestimate the potential effect of metformin in this study.

For peer review only - http://bmjopen.bmj.com/site/about/guidelines.xhtml
For peer review only

In summary, knee OA, specifically the obesity-metabolic syndrome phenotype, has limited effective treatment options. This study will provide high-quality evidence to determine whether metformin reduces knee pain in people with symptomatic knee OA with overweight or obesity over 6 months, with major clinical and public health importance for a potentially effective treatment option for knee OA to reduce knee pain and disease burden.

Acknowledgements

Ms Molly Bond, Mr Noor Abid, Mr Ashish Dinesh Nair, Dr Benjamin Sutu, Dr Talia Igel, Dr Luigi Zolio and Dr Rushab Shah have been involved in the coordination and/or execution of this study.

Contributors

Conception and design: YW, DMU, AEW, FMC. Study execution and data acquisition: YZL, YW, MME, AEW, FMC. Drafting of the manuscript: YZL, YW, FMC. Critical revision of the manuscript for important intellectual content and approval of the final manuscript: YW, DMU, MME, AEW, SH, FMC. Obtaining of funding: FMC.

Funding

The study is funded by an Investigator Grant from the National Health and Medical Research Council of Australia (NHMRC APP1194829). YZL is the recipient of National Health and Medical Research Council (NHMRC) Clinical Postgraduate Scholarship (#1133903) and Royal Australasian College of Physicians Woolcock Scholarship. YW is the recipient of NHMRC Translating Research into Practice Fellowship (APP1168185). DMU is a recipient of a NHMRC/Medical Research Future Fund (MRFF) Career Development Fellowship (Clinical Level 2 #1142809). MME is the recipient of Bangabandhu Science and Technology Fellowship from Ministry of Science and Technology, Government of the People's Republic of Bangladesh. FMC is the recipient of NHMRC Investigator Grant (APP1194829). The funders of the study had no role in the study design and conduct of the study; collection,

For peer review only - http://bmjopen.bmj.com/site/about/guidelines.xhtml

For peer review only

management, analysis and interpretation of the data; preparation, review, or approval of the manuscript; and decision to submit the manuscript for publication.

Competing interests

All authors declare no competing interests.

References

1. Vos T, Lim SS, Abbafati C, Abbas KM, Abbasi M, Abbasifard M, Abbasi-Kangevari M, Abbastabar H, Abd-Allah F, Abdelalim A *et al*: **Global burden of 369 diseases and injuries in 204 countries and territories, 1990–2019: a systematic analysis for the Global Burden of Disease Study 2019**. *The Lancet* 2020, **396**(10258):1204-1222.
2. Ferket BS, Feldman Z, Zhou J, Oei EH, Bierma-Zeinstra SMA, Mazumdar M: **Impact of total knee replacement practice: cost effectiveness analysis of data from the Osteoarthritis Initiative**. *BMJ* 2017, **356**:j1131.
3. Ackerman IN, Bohensky MA, Zomer E, Tacey M, Gorelik A, Brand CA, de Steiger R: **The projected burden of primary total knee and hip replacement for osteoarthritis in Australia to the year 2030**. *BMC Musculoskeletal Disorders* 2019, **20**(1):90.
4. van der Esch M, Knoop J, van der Leeden M, Roorda LD, Lems WF, Knol DL, Dekker J: **Clinical phenotypes in patients with knee osteoarthritis: a study in the Amsterdam osteoarthritis cohort**. *Osteoarthritis Cartilage* 2015, **23**(4):544-549.
5. Knoop J, van der Leeden M, Thorstensson CA, Roorda LD, Lems WF, Knol DL, Steultjens MP, Dekker J: **Identification of phenotypes with different clinical outcomes in knee osteoarthritis: Data from the osteoarthritis initiative**. *Arthritis Care Res (Hoboken)* 2011, **63**(11):1535-1542.
6. Thijssen E, van Caam A, van der Kraan PM: **Obesity and osteoarthritis, more than just wear and tear: pivotal roles for inflamed adipose tissue and dyslipidaemia in obesity-induced osteoarthritis**. *Rheumatology (Oxford, England)* 2015, **54**(4):588-600.
7. Puenpatom RA, Victor TW: **Increased prevalence of metabolic syndrome in individuals with osteoarthritis: an analysis of NHANES III data**. *Postgrad Med* 2009, **121**(6):9-20.
8. Berenbaum F, Eymard F, Houard X: **Osteoarthritis, inflammation and obesity**. *Curr Opin Rheumatol* 2013, **25**(1):114-118.
9. Rojas LB, Gomes MB: **Metformin: an old but still the best treatment for type 2 diabetes**. *Diabetology & metabolic syndrome* 2013, **5**(1):6.
10. Saisho Y: **Metformin and Inflammation: Its Potential Beyond Glucose-lowering Effect**. *Endocrine, metabolic & immune disorders drug targets* 2015, **15**(3):196-205.
11. Knowler WC, Fowler SE, Hamman RF, Christophi CA, Hoffman HJ, Brenneman AT, Brown-Friday JO, Goldberg R, Venditti E, Nathan DM: **10-year follow-up of diabetes incidence and weight loss in the Diabetes Prevention Program Outcomes Study**. *Lancet* 2009, **374**(9702):1677-1686.
12. Worsley R, Jane F, Robinson PJ, Bell RJ, Davis SR: **Metformin for overweight women at midlife: a double-blind, randomized, controlled trial**. *Climacteric : the journal of the International Menopause Society* 2015, **18**(2):270-277.

For peer review only - http://bmjopen.bmj.com/site/about/guidelines.xhtml

13. Lim YZ, Wang Y, Estee M, Abidi J, Udaya Kumar M, Hussain SM, Wluka AE, Little CB, Cicuttini FM: **Metformin as a potential disease-modifying drug in osteoarthritis: a systematic review of pre-clinical and human studies**. *Osteoarthritis Cartilage* 2022, **30**(11):1434-1442.
14. Li H, Ding X, Terkeltaub R, Lin H, Zhang Y, Zhou B, He K, Li K, Liu Z, Wei J *et al*: **Exploration of metformin as novel therapy for osteoarthritis: preventing cartilage degeneration and reducing pain behavior**. *Arthritis Research & Therapy* 2020, **22**(1):34.
15. Li J, Zhang B, Liu WX, Lu K, Pan H, Wang T, Oh CD, Yi D, Huang J, Zhao L *et al*: **Metformin limits osteoarthritis development and progression through activation of AMPK signalling**. *Annals of the Rheumatic Diseases* 2020, **79**(5):635-645.
16. Na HS, Kwon JY, Lee SY, Lee SH, Lee AR, Woo JS, Jung K, Cho KH, Choi JW, Lee DH *et al*: **Metformin Attenuates Monosodium-Iodoacetate-Induced Osteoarthritis via Regulation of Pain Mediators and the Autophagy-Lysosomal Pathway**. *Cells* 2021, **10**(3).
17. Moher D, Hopewell S, Schulz KF, Montori V, Gøtzsche PC, Devereaux PJ, Elbourne D, Egger M, Altman DG: **CONSORT 2010 Explanation and Elaboration: updated guidelines for reporting parallel group randomised trials**. *BMJ* 2010, **340**:c869.
18. Altman R, Asch E, Bloch D, Bole G, Borenstein D, Brandt K, Christy W, Cooke TD, Greenwald R, Hochberg M *et al*: **Development of criteria for the classification and reporting of osteoarthritis. Classification of osteoarthritis of the knee. Diagnostic and Therapeutic Criteria Committee of the American Rheumatism Association**. *Arthritis Rheum* 1986, **29**(8):1039-1049.
19. **Australian Public Assessment Report for Linagliptin / Metformin HCl. Proprietary Product Name: Trajentamet. Therapeutics Goods Administration Health Safety Regulation. September 2013**. In.
20. Pham T, van der Heijde D, Altman RD, Anderson JJ, Bellamy N, Hochberg M, Simon L, Strand V, Woodworth T, Dougados M: **OMERACT-OARSI initiative: Osteoarthritis Research Society International set of responder criteria for osteoarthritis clinical trials revisited**. *Osteoarthritis Cartilage* 2004, **12**(5):389-399.
21. Tubach F, Ravaud P, Baron G, Falissard B, Logeart I, Bellamy N, Bombardier C, Felson D, Hochberg M, van der Heijde D *et al*: **Evaluation of clinically relevant changes in patient reported outcomes in knee and hip osteoarthritis: the minimal clinically important improvement**. *Ann Rheum Dis* 2005, **64**(1):29-33.
22. Bellamy N, Buchanan WW, Goldsmith CH, Campbell J, Stitt LW: **Validation study of WOMAC: a health status instrument for measuring clinically important patient relevant outcomes to antirheumatic drug therapy in patients with osteoarthritis of the hip or knee**. *J Rheumatol* 1988, **15**(12):1833-1840.
23. Osborne RH, Hawthorne G, Lew EA, Gray LC: **Quality of life assessment in the community-dwelling elderly: validation of the Assessment of Quality of Life (AQoL) Instrument and comparison with the SF-36**. *J Clin Epidemiol* 2003, **56**(2):138-147.
24. Hochman JR, Gagliese L, Davis AM, Hawker GA: **Neuropathic pain symptoms in a community knee OA cohort**. *Osteoarthritis Cartilage* 2011, **19**(6):647-654.
25. Craig CL, Marshall AL, Sjöström M, Bauman AE, Booth ML, Ainsworth BE, Pratt M, Ekelund U, Yngve A, Sallis JF *et al*: **International physical activity questionnaire: 12-country reliability and validity**. *Med Sci Sports Exerc* 2003, **35**(8):1381-1395.
26. Kloppenburg M, Maheu E, Kraus VB, Cicuttini F, Doherty M, Dreiser RL, Henrotin Y, Jiang GL, Mandl L, Martel-Pelletier J *et al*: **OARSI Clinical Trials Recommendations: Design and conduct of clinical trials for hand osteoarthritis**. *Osteoarthritis Cartilage* 2015, **23**(5):772-786.
27. Wluka AE, Ding C, Jones G, Cicuttini FM: **The clinical correlates of articular cartilage defects in symptomatic knee osteoarthritis: a prospective study**. *Rheumatology (Oxford, England)* 2005, **44**(10):1311-1316.

For peer review only - http://bmjopen.bmj.com/site/about/guidelines.xhtml

28. Bennell KL, Paterson KL, Metcalf BR, Duong V, Eyles J, Kasza J, Wang Y, Cicuttini F, Buchbinder R, Forbes A *et al*: **Effect of Intra-articular Platelet-Rich Plasma vs Placebo Injection on Pain and Medial Tibial Cartilage Volume in Patients With Knee Osteoarthritis: The RESTORE Randomized Clinical Trial**. *Jama* 2021, **326**(20):2021-2030.
29. Cai G, Laslett LL, Aitken D, Cicuttini F, March L, Hill C, Winzenberg T, Jones G: **Zoledronic acid plus methylprednisolone versus zoledronic acid or placebo in symptomatic knee osteoarthritis: a randomized controlled trial**. *Ther Adv Musculoskelet Dis* 2019, **11**:1759720x19880054.
30. Cai G, Aitken D, Laslett LL, Pelletier JP, Martel-Pelletier J, Hill C, March L, Wluka AE, Wang Y, Antony B *et al*: **Effect of Intravenous Zoledronic Acid on Tibiofemoral Cartilage Volume Among Patients With Knee Osteoarthritis With Bone Marrow Lesions: A Randomized Clinical Trial**. *Jama* 2020, **323**(15):1456-1466.
31. Wang Y, Jones G, Hill C, Wluka AE, Forbes AB, Tonkin A, Hussain SM, Ding C, Cicuttini FM: **Effect of atorvastatin on knee cartilage volume in patients with symptomatic knee osteoarthritis: results from a randomised placebo-controlled trial**. *Arthritis & Rheumatology* 2021, **n/a**(n/a).
32. Cai G, Jones G, Cicuttini FM, Wluka AE, Wang Y, Hill C, Keen H, Antony B, Wang X, de Graaff B *et al*: **Study protocol for a randomised controlled trial of diacerein versus placebo to treat knee osteoarthritis with effusion-synovitis (DICKENS)**. *Trials* 2022, **23**(1):768.
33. Hernán MA, Lanoy E, Costagliola D, Robins JM: **Comparison of dynamic treatment regimes via inverse probability weighting**. *Basic Clin Pharmacol Toxicol* 2006, **98**(3):237-242.
34. Wluka AE, Chou L, Briggs AM, Cicuttini FM: **Understanding the needs of consumers with musculoskeletal conditions: Consumers' perceived needs of health information, health services and other non-medical services: A systematic scoping review.** In. Melbourne: MOVE muscle, bone & joint health.; 2016.
35. Park MJ, Moon SJ, Baek JA, Lee EJ, Jung KA, Kim EK, Kim DS, Lee JH, Kwok SK, Min JK *et al*: **Metformin Augments Anti-Inflammatory and Chondroprotective Properties of Mesenchymal Stem Cells in Experimental Osteoarthritis**. *Journal of Immunology* 2019, **203**(1):127-136.
36. Mohammed M, Al-Shamma K, Jassim N: **Evaluation of the Clinical use of Metformin or Pioglitazone in Combination with Meloxicam in Patients with Knee Osteoarthritis; using Knee Injury and Osteoarthritis outcome Score**. *Iraqi Journal of Pharmaceutical Sciences* 2014, **23**(2):13-23.
37. Shi Q, Wang Y, Hao Q, Vandvik PO, Guyatt G, Li J, Chen Z, Xu S, Shen Y, Ge L *et al*: **Pharmacotherapy for adults with overweight and obesity: a systematic review and network meta-analysis of randomised controlled trials**. *Lancet* 2022, **399**(10321):259-269.
38. Brebal KMM, Silveira J, Menezes RCE, Epifânio SBO, Marinho PM, Longo-Silva G: **Weight gain and changes in nutritional status of Brazilian adults after 20 years of age: a time-trend analysis (2006-2012)**. *Rev Bras Epidemiol* 2020, **23**:e200045.
39. Peeters A, Magliano DJ, Backholer K, Zimmet P, Shaw JE: **Changes in the rates of weight and waist circumference gain in Australian adults over time: a longitudinal cohort study**. *BMJ Open* 2014, **4**(1):e003667.
40. Zheng Y, Manson JE, Yuan C, Liang MH, Grodstein F, Stampfer MJ, Willett WC, Hu FB: **Associations of Weight Gain From Early to Middle Adulthood With Major Health Outcomes Later in Life**. *Jama* 2017, **318**(3):255-269.
41. Wang Y, Lombard C, Hussain SM, Harrison C, Kozica S, Brady SRE, Teede H, Cicuttini FM: **Effect of a low-intensity, self-management lifestyle intervention on knee pain in community-based young to middle-aged rural women: a cluster randomised controlled trial**. *Arthritis research & therapy* 2018, **20**(1):74.
42. Hussain SM, Ackerman IN, Wang Y, English DR, Wluka AE, Giles GG, Cicuttini FM: **Trajectories of body mass index from early adulthood to late midlife and incidence of total

For peer review only - http://bmjopen.bmj.com/site/about/guidelines.xhtml

knee arthroplasty for osteoarthritis: findings from a prospective cohort study. *Osteoarthritis Cartilage* 2023, **31**(3):397-405.
43. Katsiki N, Anagnostis P, Kotsa K, Goulis DG, Mikhailidis DP: **Obesity, Metabolic Syndrome and the Risk of Microvascular Complications in Patients with Diabetes mellitus**. *Curr Pharm Des* 2019, **25**(18):2051-2059.

Figure 1. Flowchart of trial participation

For peer review only

For peer review only - http://bmjopen.bmj.com/site/about/guidelines.xhtml

Figure 1. Flowchart of trial participation

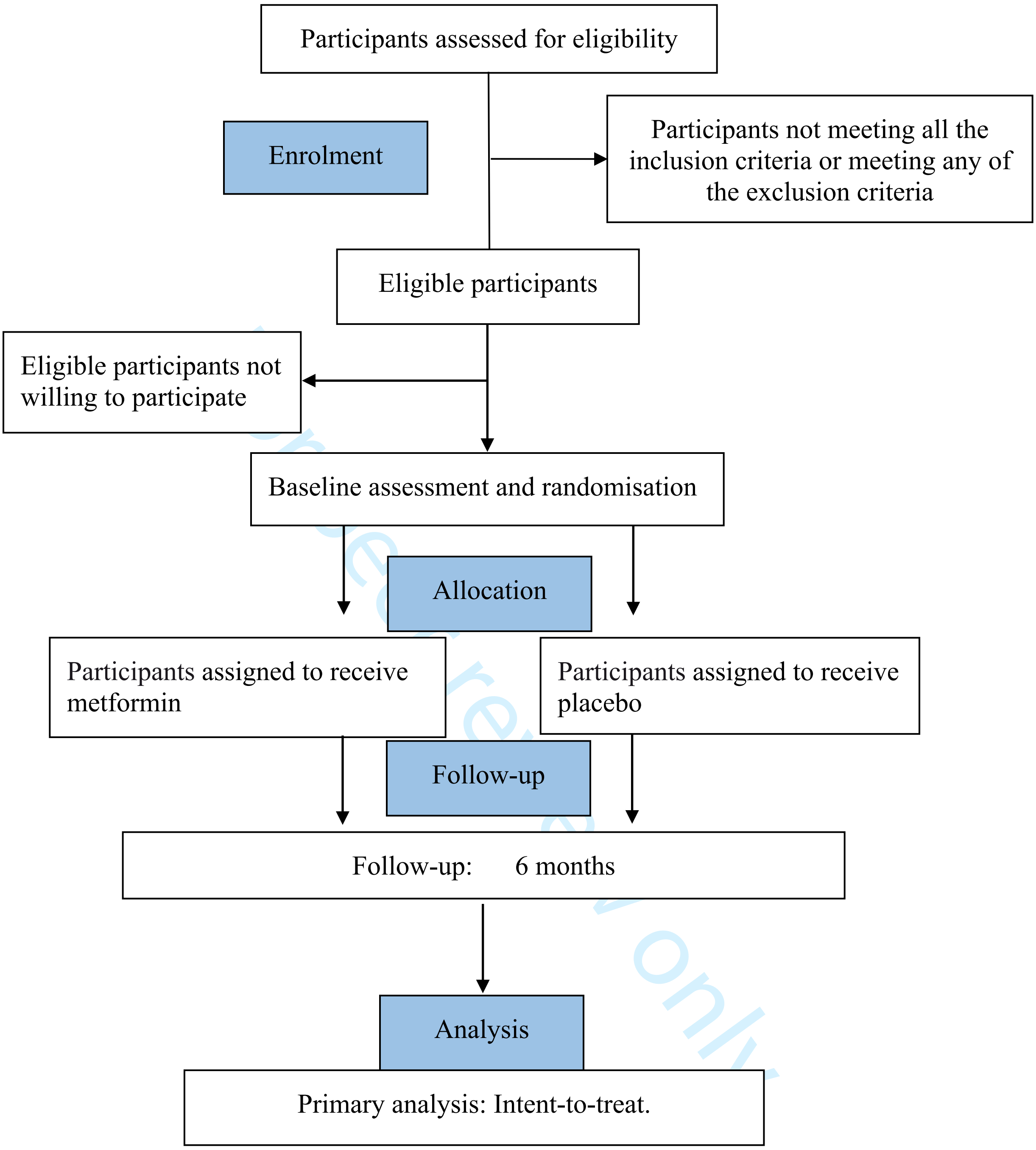

Participant Information Sheet/Consent Form – Study 1

Monash University

**Title**	Metformin for knee osteoarthritis with obesity – a randomised, double-blind, placebo-controlled trial of a potential disease modifying therapy
**Short Title**	Metformin for Knee Osteoarthritis
**Project Number**	708/20
**Project Sponsor**	Monash University
**Principal Investigators**	Professor Flavia Cicuttini, Dr Yuanyuan Wang, Professor Anita Wluka, Dr Monira Hussain
**Associate Investigators**	Ms Molly Bond, Ms Mahnuma Estee, Dr Yuan Lim, Mr Ashish Dinesh Nair, Dr Benjamin Sutu, Dr Talia Igel, Mr Noor Abid, Dr Rushab Shah, Dr Luigi Zolio, Mr Sehun Daniel Yang
**Location**	School of Public Health and Preventive Medicine, Monash University

Part 1 What does my participation involve?

1 Introduction

You are invited to take part in this research project. This is because you have osteoarthritis in your knee. The research project is testing a new treatment for knee osteoarthritis. The new treatment is metformin, a drug widely used to treat type 2 diabetes that has not been tested in osteoarthritis.

This Participant Information Sheet/Consent Form tells you about the research project. It explains the tests and treatments involved. Knowing what is involved will help you decide if you want to take part in the research.

Please read this information carefully. Ask questions about anything that you don't understand or want to know more about. Before deciding whether or not to take part, you might want to talk about it with a relative, friend or your local doctor.

Participation in this research is voluntary. If you don't wish to take part, you don't have to. You will receive the best possible care whether or not you take part.

If you decide you want to take part in the research project, you will be asked to sign the consent section. By signing it you are telling us that you:
- Understand what you have read
- Consent to take part in the research project
- Consent to have the tests and treatments that are described
- Consent to the use of your personal and health information as described.

You will be given a copy of this Participant Information and Consent Form to keep. The Participant Information and Consent Form is 13 pages long in total. Please make sure that you have all the pages.

For peer review only - http://bmjopen.bmj.com/site/about/guidelines.xhtml

2 What is the purpose of this research?

Knee osteoarthritis is a common disabling condition causing significant pain and impaired physical function. It is characterised by the loss of cartilage (the lining over the bones that gives a cushioning effect). Currently, there are no treatments that slow knee osteoarthritis progression.

Previous studies have shown that obesity is a significant risk factor for knee osteoarthritis. More than 50% of people with knee osteoarthritis are obese. Evidence suggests that obesity and obesity-associated inflammation and metabolic abnormalities such as diabetes, dyslipidaemia (abnormally elevated lipids or fats in the blood) and hypertension (high blood pressure), are potential treatment targets for those with knee osteoarthritis and obesity.

Metformin is an oral biguanide (a group of oral type 2 diabetes medications that work by preventing the production of sugar in the liver) that is a widely used and effective treatment for patients, including children, with type 2 diabetes mellitus (a condition with abnormally high levels of glucose in the blood). The main effect of metformin is to reduce blood glucose levels, resulting in sustained modest weight loss, and lowering of lipids (or fats) and inflammation. There is emerging evidence suggesting metformin may have the potential to slow the rate of knee cartilage loss and reduce pain in people with knee osteoarthritis and obesity. Metformin is well-tolerated, inexpensive and has a long history of safe clinical use.

The Metformin for Knee Osteoarthritis Study 1 is a randomised clinical trial with the aim to compare the effect of metformin with an inactive 'dummy' tablet (what is called a placebo) on knee pain and function in people with symptomatic knee osteoarthritis and overweight or obesity. If metformin is effective, it will offer a new way to improve pain and function in those with knee osteoarthritis and overweight or obesity.

Metformin has been approved in Australia (by the Australian Government's Therapeutic Goods Administration) to treat type 2 diabetes mellitus for more than 20 years. However, it is not approved to treat knee osteoarthritis. Therefore, it is an experimental treatment for knee osteoarthritis. This means that it must be tested to see if it is an effective treatment for knee osteoarthritis.

The research team conducting this study includes rheumatologists extensively experienced in the treatment of knee osteoarthritis and an endocrinologist extensively experienced with the clinical use of metformin.

A total of 102 participants will participate in this project. They will be randomly allocated into two groups, with one group receiving metformin and the other group receiving a placebo.

This research is a clinical trial being conducted by Monash University. This research has been initiated by the study investigators, Professor Flavia Cicuttini, Dr Yuanyuan Wang, Professor Anita Wluka, and Dr Monira Hussain.

This research has been funded by Monash University.

The results of this research may be used by student investigators to obtain their Honours, Masters or PhD degree.

3 What does participation in this research involve?

If you agree to participate in the study, we will ask you to do the following:

1. Participation in this trial will initially involve a screening process. You will be screened for your suitability for the study over the phone by answering simple questions about your health.

For peer review only - http://bmjopen.bmj.com/site/about/guidelines.xhtml

If you meet the inclusion criteria, a Participant Information Sheet will be mailed out to you. You will have an interview with a research assistant and a study doctor through telehealth or onsite study visit at our Clinical Trials Centre (553 St Kilda Road) Monash University, during which any question can be answered. If you agree to participate in the study, you will be asked to provide consent by signing the consent form at the end of this document (either with a pen, if you are at the study site, or electronically, if the visit is conducted by telehealth, via REDCap, a secure web platform).

You will undergo a clinical assessment and complete questionnaires.

In order to further assess your eligibility for the study, you will have a knee X-ray at I-MED Radiology and blood tests at Melbourne Pathology.

During these screening procedures, at any stage, if the researchers identify any reason that would put you at increased risk by being involved in the study, or any reason that would make it inappropriate for you to participate in the study, you will be asked not to continue in the study.

If you use telehealth option for the screening visit, you will be asked to sign the consent form electronically in REDCap (i.e. Research Electronic Data Capture, a secure web platform to capture data for clinical research) which can be accessed on a computer, mobile phone, or tablet. You will open the survey and read through the consent form (which you have already received). When you get to the bottom of the consent form, you will have the opportunity to fill in your information and 'sign' your consent by typing in your name or by utilizing REDCap's 'Signature' field type on the survey. You will select "Next Page" and a read only copy of the consent will be generated that you can review, download, and print. At the bottom of the page you will need to select "I certify that all the information in the document above is correct. I understand that clicking "Submit" will electronically sign the form and that signing this form electronically is the equivalent of signing a physical document." and "Submit". Following the telehealth screening/baseline appointment, the research assistant will post a hard copy of the signed eConsent pdf form to you to ensure you have a copy.

2. If you are eligible to participate, you will be randomly assigned to either the treatment or the control group. The term "randomised" means you will not have a choice regarding which group you are in, and you will have equal chance of receiving the active study medication or placebo.

The treatment group will receive metformin tablets (up to a dose of 2000 milligrams, 2 tablets, once daily) and the control group will receive a placebo (up to 2 tablets, once daily). A placebo is a medication with no active ingredients. It looks like the real thing but is not.

You will initially take 500 milligrams (1 tablet) per day for 2 weeks. Providing you do not experience any adverse effects, the dose will be increased by 500 milligrams (1 tablet) per day every 2 weeks until you are taking 2000 milligrams per day. You will take this dose for the rest of the study. So you will take study medication (metformin or placebo) for 6 months.

Week 1-2: 500mg (1 x 500mg tablet) per day
Week 3-4: 1000mg (2 x 500mg tablets) per day
Week 5-6: 1500mg (3 x 500mg tablets) per day
Week 7-8 and onwards: 2000mg (2 x 1000mg tablets) per day

As this is a "double-blind" trial, neither you nor the study investigator will know which treatment you are receiving until the study is completed. However, in certain circumstances the study investigator may find out which treatment you are receiving.

3. The trial will go for 6 months to examine the effect of metformin on reducing knee pain.

During the trial, you will have 2 study visits [through telehealth or attending our Clinical Trials Centre (553 St Kilda Road)]: at baseline and 6 months. Each study visit will take about 60

For peer review only - http://bmjopen.bmj.com/site/about/guidelines.xhtml

minutes. We can provide an attendance certificate signed by the research investigator for each in-person onsite study visit. We will post the study medication to your address after your baseline visit (either onsite or via telehealth).

You will also have monthly telephone interview.

4. The following measurements will occur during the study period.
   - At screening: (1) measurement or self-report of height and weight; (2) knee X-ray; and (3) blood test for kidney and liver function, fasting glucose and lipids, vitamin B12, and inflammatory biomarkers.
   - At baseline: (1) questionnaires (electronic or paper-based surveys) - information about your medical history/conditions, medication, employment, education, smoking, alcohol intake, knee pain and function, hand pain, multi-site pain, absence from paid/unpaid work, physical activity, and quality of life; (2) physical examination - height, weight, and waist circumference (if in-person visit is conducted).
   - Monthly telephone interview from month 1 to 5: knee pain and hand pain.
   - At final (6 month) follow-up: (1) questionnaires (electronic or paper-based surveys) - information about your medical history/conditions, medication, knee pain and function, hand pain, multi-site pain, absence from paid/unpaid work, physical activity, and quality of life; (2) physical examination - height, weight, and waist circumference (if in-person visit is conducted); (3) blood test for kidney and liver function, fasting glucose and lipids, vitamin B12, and inflammatory biomarkers.
   - Safety and adverse events will be recorded over the 6 months, over the phone or via face-to-face interview.
   - Compliance will be assessed by pill count at 6 months. Medication adherence will be monitored over the phone by study staff between study visits.

At baseline and final (6 months) study visits, you will be asked to complete questionnaires online in REDCap (i.e. Research Electronic Data Capture, a secure web platform to capture data for clinical research and create online databases and surveys), facilitated by the research assistant. If you prefer a hard copy, you will be provided with printed questionnaires to complete manually; the research assistant will add the data online.

The following table shows you the procedures that are done at each study visit and telephone interview.

	**Screening**	**Double-blind period**		
	**Screening/ baseline assessment**	**Randomisation**	**1-5 months**	**6 months**
**Informed consent**	X			
**Study visit (telehealth or onsite)**	X			X
**Telephone interview (monthly)**			X	
**Knee X-ray**	X			
**Blood test**	X			X
**Medical history/conditions**	X			X
**Medication**	X			X
**Employment and education**	X			
**Smoking and alcohol**	X			
**Questionnaires**				
Knee VAS	X		X	X
WOMAC	X			X

For peer review only - http://bmjopen.bmj.com/site/about/guidelines.xhtml

PainDETECT	X			X
Hand VAS	X		X	X
Multi-site pain	X			X
Absence from paid/unpaid work	X			X
AQoL	X			X
IPAQ	X			X
**Physical examination**				
Height, weight, waist circumference	X			X
**Compliance and safety (adverse events)**			X	X
**Dispense medication**		X		
VAS: visual analogue scale; WOMAC: Western Ontario and McMaster Universities Osteoarthritis Index; AQoL: Assessment of Quality of Life; AQoL: Assessment of Quality of Life; IPAQ: International Physical Activity Questionnaire				

For peer review only

This research project has been designed to make sure the researchers interpret the results in a fair and appropriate way.

There are no additional costs associated with participating in this research project, nor will you be paid. All medication, tests and medical care required as part of the research project will be provided to you free of charge.

You will be reimbursed for any reasonable travel and parking associated with the research project visit (maximum $10 per study visit). If study visits are via telehealth, you will receive a gift voucher ($10) after your baseline and final visits.

It is desirable that your local doctor be advised of your decision to participate in this research project. If you have a local doctor, we will provide a letter to your local doctor informing them of your participation in this research project.

If you choose to withdraw from this study before six months, you will be asked to attend a follow-up visit to allow collection of information regarding your health status (questionnaires and physical examination).

4 What do I have to do?

You will be required to initially take 500 milligrams (1 tablet) per day with your evening meal for 2 weeks. Providing you do not experience any adverse effects, the dose will be increased by 500 milligrams (1 tablet) per day every 2 weeks until you are taking 2000 milligrams per day. You will take this for the rest of the study (2 tablets, 1000 milligrams/tablet). So you will take study medication for a total of 6 months.

You will need to commit to taking the investigational medication regularly. You will need to attend all the study visits.

Whilst involved in this study, you can take your regular medications. We will screen for medications unsuitable for this study. You will need to record all medications and complete a questionnaire at study visits. You may not be able to donate blood while you are in the study.

You should tell your doctor and study personnel if you are taking any other medications, including any that you buy without a prescription from a pharmacy, supermarket or health food shop. Some medications may be affected by metformin or may affect how well metformin works. It is also important not to start taking any other medications during the study without talking to your doctor and research staff. If you start any new medications during the study please tell your doctor and the research staff.

For peer review only - http://bmjopen.bmj.com/site/about/guidelines.xhtml

5 Other relevant information about the research project

A total of 102 participants with symptomatic knee osteoarthritis and overweight or obesity, aged over 40 years will be recruited in Melbourne. The project will have two groups, with one group receiving metformin and the other group receiving a placebo. All participants will be followed up over 6 months to examine whether metformin has an effect on symptoms.

The project involves researchers from Monash University.

6 Do I have to take part in this research project?

Participation in any research project is voluntary. If you do not wish to take part, you do not have to. If you decide to take part and later change your mind, you are free to withdraw from the project at any stage.

If you do decide to take part, you will be given this Participant Information and Consent Form to sign and you will be given a copy to keep.

Your decision whether to take part or not to take part, or to take part and then withdraw, will not affect your routine treatment, your relationship with those treating you or your relationship with Monash University.

7 What are the alternatives to participation?

You do not have to take part in this research project to receive treatment. You can see your doctor or health care professional to discuss different treatment options for your knee osteoarthritis, such as physiotherapy and medications for pain relief. Your study doctor will discuss these options with you before you decide whether or not to take part in this research project. You can also discuss the options with your local doctor before deciding whether or not to take part in this research project.

8 What are the possible benefits of taking part?

We cannot guarantee or promise that you will receive any benefits from this research. However, some participants may experience possible benefits, such as improvement in their knee symptoms. If this study shows that metformin is effective in reducing knee pain, it may enable this treatment to be available to more people in the future.

We will inform you of any abnormal findings from knee X-rays and other tests, so that you can then consult with your doctor.

9 What are the possible risks and disadvantages of taking part?

Medical treatments often cause side effects. You may have none, some or all of the effects listed below, and they may be mild, moderate or severe. If you have any of these side effects, or are worried about them, talk with your study doctor. Your study doctor will also be looking out for side effects.

<u>Side effects of metformin</u>
Use of metformin is an established safe and well-tolerated treatment to lower blood glucose. The dose of metformin we use in this study are commonly used for type 2 diabetes mellitus. Metformin may have unwanted side effects in some people.

For peer review only - http://bmjopen.bmj.com/site/about/guidelines.xhtml

Most common side effects (affects 1 in 10 to 1 in 100 users) include:
- gastrointestinal symptoms: diarrhoea, nausea, vomiting, abdominal pain, indigestion, loss of appetite
- taste disturbance
- headache

Uncommon or rare side effects (affects 1 in 10,000 users) include:
- skin reactions: erythema, pruritus, urticarial
- lactic acidosis
- decrease of vitamin B12 absorption
- liver function test abnormalities or hepatitis resolving upon metformin discontinuation

Pregnancy risks
The effects of metformin on the unborn child and on the newborn baby are not known. Because of this, it is important that research project participants are not pregnant or breast-feeding and do not become pregnant during the course of the research project. You must not participate in the research if you are pregnant or trying to become pregnant, or breast-feeding.

If you are female and child-bearing is a possibility, you will be required to undergo a pregnancy test prior to commencing the research project.

If you are male, you should not father a child or donate sperm for at least three months after the last dose of study medication.

Both male and female participants are strongly advised to use effective contraception during the course of the research and for a period of three months after completion of the research project. You should discuss methods of effective contraception with your study doctor.

If you do become pregnant whilst participating in the research project, you should advise your study doctor immediately. Your study doctor will withdraw you from the research project and advise on further medical attention should this be necessary. You must not continue in the research if you become pregnant.

You should advise your study doctor if you father a child while participating in the research project. Your study doctor will advise on medical attention for your partner should this be necessary.

Unknown side effects
There may be side effects that the researchers do not expect or do not know about and that may be serious. Tell your study doctor immediately about any new or unusual symptoms that you get.

Many side effects go away shortly after treatment ends. However, sometimes side effects can be serious, long lasting or permanent. If a severe side effect or reaction occurs, your study doctor may need to stop your treatment. Tell the study doctor if you have any problems. Your study doctor will monitor for and discuss the best way of managing any side effects with you, should they occur.

Risks of procedures in this study
This research project involves exposure to a very small amount of radiation. As part of everyday living, everyone is exposed to naturally occurring background radiation and receives a dose of about 2 millisieverts (mSv) each year. The effective dose from this research project is less than 0.01 mSv. At this dose level, no harmful effects of radiation have been demonstrated, as any effect is too small to measure. The risk is believed to be minimal.

Have you been involved in any other research studies that involve radiation? If so, please tell us. Please keep information contained within the Patient Information and Consent Form about

For peer review only - http://bmjopen.bmj.com/site/about/guidelines.xhtml

your exposure to radiation in this study, including the radiation dose, for at least five years. You will be required to provide this information to researchers of any future research projects involving exposure to radiation.

Having blood taken may cause some discomfort, bruising, minor infection or bleeding. If this happens, it can be easily treated. Blood samples is collected by a qualified venpuncturist. We endeavour to make the collection process as simple and as stress free as possible.

If you become upset or distressed as a result of your participation in the research, the study doctor will be able to arrange for counselling or other appropriate support. Any counselling or support will be provided by qualified staff who are not members of the research project team.

10 What will happen to my test samples?

This research project involves the collection, testing and analysis of your blood samples.

The collection and testing of your blood sample is a mandatory component of the research. This is done to examine your liver function, kidney function, blood lipids, blood glucose, vitamin B12, and inflammatory biomarkers in order to assess your suitability for the study and monitor the safety and efficacy of the treatment.

Your blood samples will be re-identifiable (i.e. coded, without your name on them), and will be tested at Melbourne Pathology for the proposed blood tests. Blood samples will be destroyed after testing.

11 What if new information arises during this research project?

Sometimes during the course of a research project, new information becomes available about the treatment that is being studied. If this happens, your study doctor will tell you about it and discuss with you whether you want to continue in the research project. If you decide to withdraw, your study doctor will make arrangements for your regular health care to continue. If you decide to continue in the research project you will be asked to sign an updated consent form.

Also, on receiving new information, your study doctor might consider it to be in your best interests to withdraw you from the research project. If this happens, he/she will explain the reasons and arrange for your regular health care to continue.

12 Can I have other treatments during this research project?

Whilst you are participating in this research project, you may not be able to take some or all of the medications or treatments you have been taking for your condition or for other reasons. It is important to tell your study doctor and the study staff about any treatments or medications you may be taking, including over-the-counter medications, vitamins or herbal remedies, acupuncture or other alternative treatments. You should also tell your study doctor about any changes to these during your participation in the research project. Your study doctor will also explain to you which treatments or medications need to be stopped for the time you are involved in the research project.

It may also be necessary for you to take medication during or after the research project to address side effects or symptoms caused by participation in the study that you may have. This will be paid for by the sponsor of the study.

13 What if I withdraw from this research project?

For peer review only - http://bmjopen.bmj.com/site/about/guidelines.xhtml
For peer review only

If you decide to withdraw from the project, please notify a member of the research team before you withdraw. This notice will allow that person or the research supervisor to discuss any health risks or special requirements linked to withdrawing.

If you do withdraw your consent during the research project, the study investigator and relevant study staff will not collect additional personal information from you, although personal information already collected will be retained to ensure that the results of the research project can be measured properly and to comply with law. You should be aware that data collected by the study investigator up to the time you withdraw will form part of the research project results. If you do not want them to do this, you must tell them before you join the research project.

14 Could this research project be stopped unexpectedly?

This research project may be stopped unexpectedly for a variety of reasons. These may include reasons such as:

- Unacceptable side effects
- The drug/treatment being shown not to be effective
- The drug/treatment being shown to work and not requiring further testing

15 What happens when the research project ends?

At the completion of the trial, if you wish to know whether you received metformin or the placebo, please contact the research personnel for further information.

We will send you a follow-up letter to inform you of the findings of the study.

Metformin or placebo will be provided to you during the trial for a 6 month period at no cost. Once the trial has finished we will not be able to continue to provide this treatment to you. However, if your doctor is in agreement with continuing the treatment after the trial, he/she can provide you with a prescription for metformin. From this point you will need to cover the cost of your medication.

Part 2 How is the research project being conducted?

16 What will happen to information about me?

By signing the consent form you consent to the study investigator and relevant research staff collecting and using personal information about you for the research project. Any information obtained in connection with this research project that can identify you will remain confidential.

Monash REDCap will be used for collection and storage of data. The REDCap is password protected and access restricted to study investigators. The data we collect or use will be individually identifiable or re-identifiable (i.e. coded which means that the data we collect from you will be directly linked to your study ID number. A study ID number will be allocated to each participant so that their name is not on all data.). All electronic data will be kept in password-protected databases, separate from identifying information. The completed eConsent PDFs are stored in REDCap in a File Repository under “PDF Survey Archive”. Where data is collected on paper, this will be kept in locked filing cabinets with restricted key access, at the School of Public Health and Preventive Medicine, Monash University.

Your knee x-ray will be name-identified in accordance with standard clinical practice, and will be stored securely and indefinitely in the I-MED Radiology database and password-protected databases at the School of Public Health and Preventive Medicine, Monash University. Access to data will be limited to the principal and associate investigators and support staff only. Data

For peer review only - http://bmjopen.bmj.com/site/about/guidelines.xhtml

transfer will occur so that the final dataset with re-identifiable (i.e. coded) data can be accessed by all the chief investigators of the study. Identifiable information will not be released to anyone outside the research team.

Your information will only be used for the purpose of this research project and it will only be disclosed with your permission, except as required by law.

By signing the consent form you consent to the study investigator using your data collected for this project for extended related research. Your data collected for this project may be combined with data collected from other studies for meta-analyses. In this case only re-identifiable data will be used.

Information from questionnaires and physical examinations will be retained for at least 15 years upon completion of the study. This research project does not involve the establishment of a databank.

It is necessary that your local doctor be advised of your decision to participate in this research project. By signing the consent section, you agree to your local doctor being notified of your decision to participate in this research project.

It is anticipated that the results of this research project will be published and presented in a variety of forums. In any publication, report, or presentation, information will be provided in such a way that you cannot be identified, except with your permission. This confidentiality will be maintained by presenting aggregate data.

Should any sharing of data be considered (e.g. for combining data with other studies), then data sharing will only involve re-identified (i.e. coded) data, i.e. data that is shared will not include identifiable information. In the event where personal information (e.g. name, date of birth) may need to be shared (e.g. data linkage), we will contact you or your guardian for consent for data sharing.

In accordance with relevant Australian and Victorian privacy and other relevant laws, you have the right to request access to your information collected and stored by the research team. You also have the right to request that any information with which you disagree be corrected. Please contact the study team member named at the end of this document if you would like to access your information.

If you are not satisfied with how your personal information has been handled (as laid out in the Privacy Act, 1988), then you can make a complaint to the Office of the Australian Information Commissioner (OAIC). Please refer to http://www.oaic.gov.au/privacy/privacy-complaints for more information.

17 Complaints and compensation

If you suffer any injuries or complications as a result of this research project, you should contact the study team as soon as possible and you will be assisted with arranging appropriate medical treatment. If you are eligible for Medicare, you can receive any medical treatment required to treat the injury or complication, free of charge, as a public patient in any Australian public hospital.

18 Who is organising and funding the research?

This research project is being conducted at Melbourne by Professor Flavia Cicuttini, Dr Yuanyuan Wang, Professor Anita Wluka, and Dr Monira Hussain, and is being funded by Monash University.

For peer review only - http://bmjopen.bmj.com/site/about/guidelines.xhtml

By taking part in this research project you agree that data generated from this project may be provided to Monash University. Monash University may directly or indirectly benefit financially from knowledge acquired through analysis of your data.

You will not benefit financially from your involvement in this research project even if, for example, knowledge acquired from analysis of your data prove to be of commercial value to Monash University.

In addition, if knowledge acquired through this research leads to discoveries that are of commercial value to Monash University, the study investigators or their institutions, there will be no financial benefit to you or your family from these discoveries.

No member of the research team will receive a personal financial benefit from your involvement in this research project (other than their ordinary wages).

19 Who has reviewed the research project?

All research in Australia involving humans is reviewed by an independent group of people called a Human Research Ethics Committee (HREC). The ethical aspects of this research project have been approved by the Alfred Hospital Ethics Committee.

This project will be carried out according to the *National Statement on Ethical Conduct in Human Research (2007)*. This statement has been developed to protect the interests of people who agree to participate in human research studies.

20 Further information and who to contact

The person you may need to contact will depend on the nature of your query.

If you want any further information concerning this project or if you have any medical problems which may be related to your involvement in the project (for example, any side effects), you can contact the principal investigator:

Professor Flavia Cicuttini (Monash University): 9903 0158, flavia.cicuttini@monash.edu

Clinical contact person

Name	Professor Flavia Cicuttini	
Position	Head Rheumatology, Alfred Hospital<br>Head Musculoskeletal Unit, Monash University	Alfred Hospital Rheumatology Registrar (outside office hours)
Telephone	9903 0158	9076 2000
Email	flavia.cicuttini@monash.edu	

Project team contact information

Name	
Position	Project officer
Telephone	9903 0553
Email	jointstudy@monash.edu

For matters relating to research at the site at which you are participating, the details of the local site complaints person are:

Position	Complaints Officer (Alfred Health)

For peer review only - http://bmjopen.bmj.com/site/about/guidelines.xhtml

Telephone	03 9076 3619
Email	research@alfred.org.au

Complaints contact person

Position	Complaints Officer (Monash University)
Telephone	03 9905 2052
Email	muhrec@monash.edu

If you have any complaints about any aspect of the project, the way it is being conducted or any questions about being a research participant in general, then you may contact:

Reviewing HREC approving this research and HREC Executive Officer details

Reviewing HREC name	Alfred Hospital Ethics Committee
Position	HREC Executive Officer
Telephone	03 9076 3619
Email	research@alfred.org.au

For peer review only

For peer review only - http://bmjopen.bmj.com/site/about/guidelines.xhtml

Consent Form

**Title**	Metformin for knee osteoarthritis with obesity – a randomised, double-blind, placebo-controlled trial of a potential disease modifying therapy
**Short Title**	Metformin for Knee Osteoarthritis
**Project Number**	708/20
**Project Sponsor**	Monash University
**Principal Investigators**	Professor Flavia Cicuttini, Dr Yuanyuan Wang, Professor Anita Wluka, Dr Monira Hussain
**Associate Investigators**	Ms Molly Bond, Ms Mahnuma Estee, Dr Yuan Lim, Mr Ashish Dinesh Nair, Dr Benjamin Sutu, Dr Talia Igel, Mr Noor Abid, Dr Rushab Shah, Dr Luigi Zolio, Mr Sehun Daniel Yang
**Location**	School of Public Health and Preventive Medicine, Monash University

Consent Agreement

I have read the Participant Information Sheet or someone has read it to me in a language that I understand.

I understand the purposes, procedures and risks of the research described in the project.

I give permission for my doctors, other health professionals, hospitals or laboratories outside this hospital to release information to Monash University concerning my disease and treatment for the purposes of this project. I understand that such information will remain confidential.

I have had an opportunity to ask questions and I am satisfied with the answers I have received.

I freely agree to participate in this research project as described and understand that I am free to withdraw at any time during the study without affecting my future health care.

I understand that I will be given a signed copy of this document to keep.

I understand that, if I decide to discontinue the study treatment, I may be asked to attend follow-up visits to allow collection of information regarding my health status.

❒ eConsent was obtained using telehealth via Monash University REDCap.

Declaration by Participant – for participants who have read the information

Name of Participant (please print) ____________________

Signature ____________________ Date ____________________

Declaration by Study Doctor/Senior Researcher†

I have given a verbal explanation of the research project, its procedures and risks and I believe that the participant has understood that explanation.

Name of Study Doctor/
Senior Researcher† (please print) ____________________

Signature ____________________ Date ____________________

† A senior member of the research team must provide the explanation of, and information concerning, the research project. Note: All parties signing the consent section must date their own signature.

For peer review only - http://bmjopen.bmj.com/site/about/guidelines.xhtml

SPIRIT

STANDARD PROTOCOL ITEMS: RECOMMENDATIONS FOR INTERVENTIONAL TRIALS

SPIRIT 2013 Checklist: Recommended items to address in a clinical trial protocol and related documents*

Section/item	Item No	Description	Page
**Administrative information**			
Title	1	Descriptive title identifying the study design, population, interventions, and, if applicable, trial acronym	**1**
Trial registration	2a	Trial identifier and registry name. If not yet registered, name of intended registry	2, 5
	2b	All items from the World Health Organization Trial Registration Data Set	NA
Protocol version	3	Date and version identifier	NA
Funding	4	Sources and types of financial, material, and other support	1
Roles and responsibilities	5a	Names, affiliations, and roles of protocol contributors	1, 13
	5b	Name and contact information for the trial sponsor	1, 13
	5c	Role of study sponsor and funders, if any, in study design; collection, management, analysis, and interpretation of data; writing of the report; and the decision to submit the report for publication, including whether they will have ultimate authority over any of these activities	1, 13
	5d	Composition, roles, and responsibilities of the coordinating centre, steering committee, endpoint adjudication committee, data management team, and other individuals or groups overseeing the trial, if applicable (see Item 21a for data monitoring committee)	NA
**Introduction**			
Background and rationale	6a	Description of research question and justification for undertaking the trial, including summary of relevant studies (published and unpublished) examining benefits and harms for each intervention	4
	6b	Explanation for choice of comparators	4
Objectives	7	Specific objectives or hypotheses	4,5
Trial design	8	Description of trial design including type of trial (eg, parallel group, crossover, factorial, single group), allocation ratio, and framework (eg, superiority, equivalence, noninferiority, exploratory)	4

For peer review only

Methods: Participants, interventions, and outcomes

Study setting	9	Description of study settings (eg, community clinic, academic hospital) and list of countries where data will be collected. Reference to where list of study sites can be obtained	5
Eligibility criteria	10	Inclusion and exclusion criteria for participants. If applicable, eligibility criteria for study centres and individuals who will perform the interventions (eg, surgeons, psychotherapists)	5,6
Interventions	11a	Interventions for each group with sufficient detail to allow replication, including how and when they will be administered	7
	11b	Criteria for discontinuing or modifying allocated interventions for a given trial participant (eg, drug dose change in response to harms, participant request, or improving/worsening disease)	7
	11c	Strategies to improve adherence to intervention protocols, and any procedures for monitoring adherence (eg, drug tablet return, laboratory tests)	7
	11d	Relevant concomitant care and interventions that are permitted or prohibited during the trial	7
Outcomes	12	Primary, secondary, and other outcomes, including the specific measurement variable (eg, systolic blood pressure), analysis metric (eg, change from baseline, final value, time to event), method of aggregation (eg, median, proportion), and time point for each outcome. Explanation of the clinical relevance of chosen efficacy and harm outcomes is strongly recommended	8
Participant timeline	13	Time schedule of enrolment, interventions (including any run-ins and washouts), assessments, and visits for participants. A schematic diagram is highly recommended (see Figure)	16
Sample size	14	Estimated number of participants needed to achieve study objectives and how it was determined, including clinical and statistical assumptions supporting any sample size calculations	9, 10
Recruitment	15	Strategies for achieving adequate participant enrolment to reach target sample size	5

Methods: Assignment of interventions (for controlled trials)

Allocation:

Sequence generation	16a	Method of generating the allocation sequence (eg, computer-generated random numbers), and list of any factors for stratification. To reduce predictability of a random sequence, details of any planned restriction (eg, blocking) should be provided in a separate document that is unavailable to those who enrol participants or assign interventions	6

Allocation concealment mechanism	16b	Mechanism of implementing the allocation sequence (eg, central telephone; sequentially numbered, opaque, sealed envelopes), describing any steps to conceal the sequence until interventions are assigned	6
Implementation	16c	Who will generate the allocation sequence, who will enrol participants, and who will assign participants to interventions	6
Blinding (masking)	17a	Who will be blinded after assignment to interventions (eg, trial participants, care providers, outcome assessors, data analysts), and how	6
	17b	If blinded, circumstances under which unblinding is permissible, and procedure for revealing a participant's allocated intervention during the trial	6,7
**Methods: Data collection, management, and analysis**			
Data collection methods	18a	Plans for assessment and collection of outcome, baseline, and other trial data, including any related processes to promote data quality (eg, duplicate measurements, training of assessors) and a description of study instruments (eg, questionnaires, laboratory tests) along with their reliability and validity, if known. Reference to where data collection forms can be found, if not in the protocol	7,8
	18b	Plans to promote participant retention and complete follow-up, including list of any outcome data to be collected for participants who discontinue or deviate from intervention protocols	8
Data management	19	Plans for data entry, coding, security, and storage, including any related processes to promote data quality (eg, double data entry; range checks for data values). Reference to where details of data management procedures can be found, if not in the protocol	10, 11
Statistical methods	20a	Statistical methods for analysing primary and secondary outcomes. Reference to where other details of the statistical analysis plan can be found, if not in the protocol	10
	20b	Methods for any additional analyses (eg, subgroup and adjusted analyses)	10
	20c	Definition of analysis population relating to protocol non-adherence (eg, as randomised analysis), and any statistical methods to handle missing data (eg, multiple imputation)	10
**Methods: Monitoring**			
Data monitoring	21a	Composition of data monitoring committee (DMC); summary of its role and reporting structure; statement of whether it is independent from the sponsor and competing interests; and reference to where further details about its charter can be found, if not in the protocol. Alternatively, an explanation of why a DMC is not needed	7

For peer review only - http://bmjopen.bmj.com/site/about/guidelines.xhtml

	21b	Description of any interim analyses and stopping guidelines, including who will have access to these interim results and make the final decision to terminate the trial	7
Harms	22	Plans for collecting, assessing, reporting, and managing solicited and spontaneously reported adverse events and other unintended effects of trial interventions or trial conduct	7
Auditing	23	Frequency and procedures for auditing trial conduct, if any, and whether the process will be independent from investigators and the sponsor	NA
**Ethics and dissemination**			
Research ethics approval	24	Plans for seeking research ethics committee/institutional review board (REC/IRB) approval	5
Protocol amendments	25	Plans for communicating important protocol modifications (eg, changes to eligibility criteria, outcomes, analyses) to relevant parties (eg, investigators, REC/IRBs, trial participants, trial registries, journals, regulators)	5
Consent or assent	26a	Who will obtain informed consent or assent from potential trial participants or authorised surrogates, and how (see Item 32)	5
	26b	Additional consent provisions for collection and use of participant data and biological specimens in ancillary studies, if applicable	NA
Confidentiality	27	How personal information about potential and enrolled participants will be collected, shared, and maintained in order to protect confidentiality before, during, and after the trial	11
Declaration of interests	28	Financial and other competing interests for principal investigators for the overall trial and each study site	13
Access to data	29	Statement of who will have access to the final trial dataset, and disclosure of contractual agreements that limit such access for investigators	13
Ancillary and post-trial care	30	Provisions, if any, for ancillary and post-trial care, and for compensation to those who suffer harm from trial participation	7
Dissemination policy	31a	Plans for investigators and sponsor to communicate trial results to participants, healthcare professionals, the public, and other relevant groups (eg, via publication, reporting in results databases, or other data sharing arrangements), including any publication restrictions	11
	31b	Authorship eligibility guidelines and any intended use of professional writers	13
	31c	Plans, if any, for granting public access to the full protocol, participant-level dataset, and statistical code	14

For peer review

Appendices

Informed consent materials	32	Model consent form and other related documentation given to participants and authorised surrogates	https://www.monash.edu/medicine/sphpm/units/musculoskeletal-epidemiology/join-our-studies
Biological specimens	33	Plans for collection, laboratory evaluation, and storage of biological specimens for genetic or molecular analysis in the current trial and for future use in ancillary studies, if applicable	NA

*It is strongly recommended that this checklist be read in conjunction with the SPIRIT 2013 Explanation & Elaboration for important clarification on the items. Amendments to the protocol should be tracked and dated. The SPIRIT checklist is copyrighted by the SPIRIT Group under the Creative Commons "Attribution-NonCommercial-NoDerivs 3.0 Unported" license.

For peer review only
